# Genomic Profiling of Collaborative Cross Founder Mice Infected with Respiratory Viruses Reveals Novel Transcripts and Infection-Related Strain-Specific Gene and Isoform Expression

**DOI:** 10.1534/g3.114.011759

**Published:** 2014-06-05

**Authors:** Hao Xiong, Juliet Morrison, Martin T. Ferris, Lisa E. Gralinski, Alan C. Whitmore, Richard Green, Matthew J. Thomas, Jennifer Tisoncik-Go, Gary P. Schroth, Fernando Pardo-Manuel de Villena, Ralph S. Baric, Mark T. Heise, Xinxia Peng, Michael G. Katze

**Affiliations:** * Department of Microbiology, School of Medicine, University of Washington, Seattle, Washington; † Pacific Northwest Regional Center of Excellence for Biodefense and Emerging Infectious Diseases Research, Portland, Oregon; ‡ Department of Epidemiology, University of North Carolina-Chapel Hill, Chapel Hill, North Carolina; § Department of Genetics, University of North Carolina-Chapel Hill, Chapel Hill, North Carolina; ** Illumina, Inc., San Diego, California

**Keywords:** RNA-seq, mouse transcriptome annotation, isoform differential expression, collaborative cross, viral infection

## Abstract

Genetic variation between diverse mouse species is well-characterized, yet existing knowledge of the mouse transcriptome comes largely from one mouse strain (C57BL/6J). As such, it is unlikely to reflect the transcriptional complexity of the mouse species. Gene transcription is dynamic and condition-specific; therefore, to better understand the mouse transcriptional response to respiratory virus infection, we infected the eight founder strains of the Collaborative Cross with either influenza A virus or severe acute respiratory syndrome coronavirus and sequenced lung RNA samples at 2 and 4 days after infection. We found numerous instances of transcripts that were not present in the C57BL/6J reference annotation, indicating that a nontrivial proportion of the mouse genome is transcribed but poorly annotated. Of these novel transcripts, 2150 could be aligned to human or rat genomes, but not to existing mouse genomes, suggesting functionally conserved sequences not yet recorded in mouse genomes. We also found that respiratory virus infection induced differential expression of 4287 splicing junctions, resulting in strain-specific isoform expression. Of these, 59 were influenced by strain-specific mutations within 2 base pairs of key intron–exon boundaries, suggesting *cis*-regulated expression. Our results reveal the complexity of the transcriptional response to viral infection, previously undocumented genomic elements, and extensive diversity in the response across mouse strains. These findings identify hitherto unexplored transcriptional patterns and undocumented transcripts in genetically diverse mice. Host genetic variation drives the complexity and diversity of the host response by eliciting starkly different transcriptional profiles in response to a viral infection.

Genetic diversity is an important consideration when assessing the clinical applicability of data gained from mouse studies (Churchill 2014). This has led to the development of genetic diversity resources such as the Collaborative Cross (CC), a group of genetically diverse recombinant inbred (RI) mice descending from eight founder strains: five laboratory strains (C57BL/6J, A/J, NOD/ShiLtJ, NZO/HILtJ, and 129S1/SvImJ) and three wild-derived strains (CAST/EiJ, PWK/PhJ, and WSB/EiJ). The complex phenotypes that are exhibited by CC mice are rooted in their genetic variation (Ferris *et al.* 2013; Aylor *et al.* 2011; Durrant *et al.* 2011; Kelada *et al.* 2012) and, as such, these mice are valuable tools for genetic mapping and systems research. While genetic variations between commonly used mouse strains have been characterized to a large extent (Collaborative Cross Consortium 2012; Keane *et al.* 2011), the transcriptional landscape of genetically diverse mice has received little attention, with the Mouse ENCODE project focusing exclusively on the *Mus musculus domesticus* C57BL/6J strain (Mouse ENCODE Consortium *et al.* 2012). Although some mouse strains are closely related, such as the classical laboratory inbred strains C57BL/6J and A/J, wild-derived strains such as PWK/PhJ and CAST/EiJ are more genetically variable and, in fact, represent the *M. musculus castaneus* and *M. musculus musculus* subspecies, respectively (Collaborative Cross Consortium 2012). Wild-derived inbred strains can harbor tens of millions of SNPs, hundreds of thousands of small indels, and tens of thousands of structural variations compared with the reference mouse genome (Keane *et al.* 2011). This means that the genetic variation that exists among a few mouse strains can be as large as the genetic variation that exists among the 1092 humans sequenced by the 1000 Genomes Project (McVean *et al.* 2012). In fact, there are more large structural differences between the CAST/EiJ strain and the mouse reference annotation than among all of the humans in the 1000 Genomes Project—25,000 *vs.* 14,000. Given these enormous genetic differences, and the fact that the mouse reference annotation is based on the C57BL/6J strain, it is likely that there are numerous new transcriptional elements with unknown functions that have yet to be recorded in the reference annotation.

Our understanding of transcription has been steadily augmented since the advent of RNA-seq (Mouse ENCODE Consortium *et al.* 2012; ENCODE Project Consortium *et al.* 2012). More than half of the human genome is transcribed, yet no single cell line exhibits more than 60% of the total human transcriptome (Djebali *et al.* 2012). Many transcripts have been found in intergenic regions, particularly the long intergenic noncoding RNAs (lincRNAs) (Derrien *et al.* 2012). This suggests both transcriptional complexity and transcriptional diversity. In addition, transcription is dynamic and condition-dependent and is impacted by genetic variations (Montgomery *et al.* 2010; Pickrell *et al.* 2010). Therefore, the large genetic diversity of laboratory mouse strains leads to more transcriptional diversity than is reflected in the reference annotation. For example, the interferon-inducible *Mx1* gene is one of the first host genes that was linked to influenza A virus (IAV) pathogenesis, and it limits disease by inhibiting viral replication (Staeheli *et al.* 1988; Tumpey *et al.* 2007). *Mx1* is naturally polymorphic; the *Mx1* gene has three exons that are absent in C57BL/6J mice but present in CAST/EiJ mice (Ferris *et al.* 2013). As such, these exons are not recorded in the reference annotation.

The absence of alternative transcripts in the reference annotation has implications for understanding disease pathogenesis. For example, transcriptional control mechanisms, such as alternative promoter and terminator usage, are prevalent in genes associated with neurological disease (Pal *et al.* 2011); and novel alternatively spliced transcripts have been linked to lung fibrosis development (Deng *et al.* 2013). Alternative transcription is also important in the host response to virus infection. Transcriptional start site (TSS) selections and posttranscriptional modification to host transcripts have been linked to dengue virus virulence (Sessions *et al.* 2013). Long noncoding RNAs have been linked to activation and repression of the inflammatory response to a TLR2 ligand (Carpenter *et al.* 2013; Chang *et al.* 2011) and to inflammatory signaling (Rapicavoli *et al.* 2013), and unique noncoding RNA signatures have been associated with lethality of severe acute respiratory syndrome coronavirus (SARS-CoV) (Peng *et al.* 2010). As genetically diverse mouse populations such as the CC mice become increasingly used for expression quantitative trait loci (eQTL) (Bottomly *et al.* 2012) and transcriptomic studies (Pommerenke *et al.* 2012), the need for a well-annotated mouse transcriptome increases commensurately.

The goal of this study was to characterize the transcriptional landscape of genetically diverse mice in response to respiratory viral infection and to identify new transcriptional elements that have yet to be recorded in the reference annotation. To this end, we sequenced day 2 and day 4 lung samples from the eight founder strains of the CC project that had been mock-infected or infected with mouse-adapted strains of two important respiratory pathogens, IAV or SARS-CoV. We built a custom bioinformatics pipeline to filter the results of *de novo* assembly by focusing on intergenic novel transcripts and aligning them with mouse expressed sequence tag (EST) and clone databases and with rat and human genomes. We found that many novel transcripts with potential roles in the host response to viral infection existed outside of the reference annotation. A smaller number of novel transcripts could not be aligned with the reference mouse genome or the pseudogenomes generated from the reference genome and genome sequencing of the founder mice, further demonstrating missing information in existing mouse genome sequence data. We found strain-specific differentially expressed (DE) isoforms between samples from virus-infected and mock-infected animals, some of which were likely *cis*-regulated by genetic variations near intron–exon boundaries. Strain-specificity was also observed in the functional enrichment of DE genes, with one subset of DE genes closely reflecting the underlying genetic variations and another subset suggesting differences in the immune response to viral infection among the different founder strains.

## Materials and Methods

### Animal infection and RNA extraction

Female mice of the eight CC founder strains [A/J (AJ), C57BL/6J (C57BL6J), 129S1/SvImJ (129S1), NOD/ShiLtJ (NOD), NZO/HILtJ (NZO), CAST/EiJ (CAST), PWK/PhJ (PWK), and WSB/EiJ (WSB)] were bred in-house at the University of North Carolina at Chapel Hill and were infected when they were between 8 and 16 weeks old. They were kept in a pathogen-free environment until infection. After being lightly anesthetized, the mice received 50 µL of PBS and were infected with 5 × 10^2^ PFU of PR8 (Schickli *et al.* 2001) or 10^5^ PFU of MA15. Mock-infected animals received 50 µL of PBS. The animals were monitored daily for their morbidity. At day 2 and day 4 after infection, three of the infected mice and two mock-infected animals were killed and their lungs were collected. All experiments were approved by the University of North Caroline Chapel Hill Institutional Animal Care and Use Committee.

RNA was extracted from lung tissue homogenate with the miRNeasy mini kit with on-column DNase I treatment (Qiagen). RNA concentration was quantified on an ND-2000c UV-Vis spectrophotometer (Nanodrop, Wilmington, DE) and controlled for integrity and purity on a capillary electrophoresis system (Agilent 2100 Bioanalyzer; Agilent Technologies, Santa Clara, CA).

### Strand-specific total RNA library preparation and sequencing

The total RNA-Seq library was constructed using the Illumina TruSeq Stranded Total RNA Sample Prep Kit with Ribo-Zero Gold (catalog #RS-122-2201), which targets RNA for cDNA synthesis. This kit uses Ribo-Zero to remove rRNA prior to library preparation. The libraries were quality-controlled for appropriate mass and insert size (also for evidence of DNA contamination) and quantitated using the BioAnalyzer 2100 system and qPCR (Kapa Biosystems, Woburn, MA). Sequencing was performed initially with the Illumina HiSeq 2000 system using HiSeq version 3 sequencing reagents. Some additional data were generated using the Genome Analyzer IIx system using GA version 5 sequencing reagents to supplement the number of reads up to a minimum level for all libraries. The libraries were clonally amplified on a cluster generation station using Illumina HiSeq version 3 and GA version 4 cluster generation reagents to achieve a target density of approximately 700,000/mm^2^ in a single channel of a flow cell. Image analysis, base calling, and error estimation were performed using Illumina Analysis Pipeline (version 2.8). RTA analysis (RTA version 1.13 for the HiSeq and RTA version 1.9 for the GAIIx) for both the HiSeq and Genome Analyzer reproduces base-calling and quality thresholds because both machines use similar SBS chemistry. The raw reads have been uploaded with accession number GSE52405 (Josset *et al.* 2014).

### Quality control and alignment

Read quality was checked with FASTQC Illumina Sequence Analysis Viewer (SAV) and reads were trimmed to remove primers to length 97 using in-house custom scripts. Additional quality control was performed at the individual-run level by comparing log-read counts between replicates and between mocks of the same strain. Evidence of PCR artifacts and DNA contamination were not visible during sequencing quality control and alignment. One sequencing run was deleted because of obvious discrepancy with other replicates. Viral infection was monitored by checking viral reads, and sample mice for which no viral reads could be detected were removed from further analysis.

Prior to aligning to host genomes, short reads were aligned to ribosomal sequences using Bowtie1. Any read aligned to ribosomes was removed. In the next step, the remaining reads were mapped to viral genomes, PR8 and MA15, and any mapped read was similarly removed. The number of reads removed at this step was recorded as viral read counts. The leftover reads were then aligned to pseudogenomes downloaded from the University of North Carolina compgene web site, version 0.1, using STAR version 2.2.0c. Alignment was also performed on the reference genome (version mm9) as a comparison.

### Transcript discovery

Novel transcript discovery had to take into account the existence of the reference annotation and reference genome and strain-adapted pseudogenomes. As a result, we first downloaded the reference annotation’s sequences from Ensembl’s ftp server using version GRCm38, including both coding transcripts and noncoding transcripts, totaling 92,179 transcripts. Transcripts not yet placed on chromosomes but on scaffolds were extracted and kept separately for later processing. Then, the sequence of the transcripts was mapped to the pseudogenomes developed by the University of North Carolina Systems Genetics group (http://csbio.unc.edu/CCstatus/index.py?run=Pseudo), version 0.1, using GMAP version 2013-08-14 (Wu and Watanabe 2005). The command line used was “-B 5 –t 10 –O–split-output–format=gff3_gene,” in addition to the genomes' location. The genome indices were built using a companion program of GMAP (“gmap_build”) with default options.

The mapping of RNA-seq short reads to different strains’ genomes resulted in two sets, one in which reads were mapped to genomes and another in which reads were not mapped. *De novo* transcript assembly using unmapped reads was performed with Trinity. Transcript assembly using mapped reads was performed using Cufflinks version 2.0.2 (Trapnell *et al.* 2012). The command line used was “–library-type fr-firststrand.” The Cufflinks’ output annotation file was then compared with adapted annotation to filter out all transcripts overlapping known transcripts or, in other words, to extract intergenic transcripts. The output from Trinity was first filtered by mapping to viral and ribosomal genomes and then mapped to genomes to obtain transcripts mapped to existing genomes and truly *de novo* transcripts unmappable to existing genomes; the mapping was performed using GMAP with the same command line options as above. Single-exon transcripts were oriented to positive strand or negative strand, depending on which strand resulted in higher read counts. The read counts were generated by htseq-count, part of HTSeq software affiliated with the DESeq package (Anders and Huber 2010). The command line option was default except “–stranded=reverse.” Afterward, additional filtering for intergenic transcripts was performed using cuffcompare with “reference” annotation set using “-r” option. The intergenic transcripts from the output of Cufflinks and Trinity were then merged using cuffmerge with default options. The *de novo* transcripts from Trinity’s output were compacted using the sequencing clustering program cd-hit (http://www.bioinformatics.org/cd-hit/) version 4.5.4 and option “’-c 0.95 -n 9 -g 1 -M 0 -T 20.” Then, the sequences of novel transcripts were matched against all known human or mouse DNA or RNA sequences downloaded from NCBI. The program used was “blastn” version 2.2.28 downloaded and run locally. The command line options used were “-db nt -perc_identity 90 -num_threads 20 -outfmt “6 qseqid sseqid pident length qlen slen mismatch gapopen staxids salltitles””. The database of sequences was downloaded to a companion program “update_blastdb.pl” downloaded along with blastn.

### Differential expression and functional analysis

The read counts were generated using htseq-count version 0.5.3p9. The command line options were as specified above. Differential expression analysis was performed using edgeR software version 3.2.4 in R version 3.0.1. All statistical tests were performed using GLM-related functions. Normalization was performed by function "calcNormFactors" in edgeR. Filtering of genes was performed based on whether a gene’s read counts had at least two samples with one count per million reads. The counts-per-million is a normalized read count returned by function “cpm” in the edgeR package. The design matrix was generated using “MOCK” as the baseline target. Variance estimation was by a sequence of steps, “estimateGLMCommonDisp,” “estimateGLMTrendedDisp,” “estimateGLMTagwiseDisp.” The statistical test was performed by “glmLRT” function and fitting of the negative binomial model was performed by “glmFit.” The cut-off criterion for statistical significance was false-discovery rate of 0.01 and log-fold change absolute value of at least 1.

The primary functional analysis was performed with Ingenuity Pathway Analysis (IPA) from Ingenuity Systems Inc. (http://www.ingenuity.com). The output of edgeR was fed to IPA and filtering of significant genes was performed within IPA to give a proper background of genes for enrichment analysis. GO term enrichment analysis was provided by “GOSim” and “topGO” from Biodconductor with version 1.4.0 and 2.14.0, respectively. The enrichment was computed based on a background of DE genes to reduce bias.

## Results

### Genetic background determines viral load and disease severity in response to IAV and SARS-CoV infection

The eight founder strains of the CC were infected with either 5 × 10^2^ PFU of mouse-adapted influenza A/Puerto Rico/8/34 virus (PR8) or 1 × 10^5^ PFU of mouse-adapted SARS-CoV (MA15), and lung samples were collected at day 2 and day 4 after infection, with three replicates at each time point. Time-matched lung samples from mock-infected animals were also collected, resulting in a total of 124 lung samples. Total RNA was then sequenced using an Illumina HiSeq with a stranded library preparation protocol that generated 2 × 100 nt paired-ends reads. Each sample was ensured to have at least 30 million reads and, on average, there were 48 million reads per sample. We then verified the sequence quality and filtered out ribosomal RNA reads and viral reads. The viral read counts varied among the founder strains, suggesting that the genotypes of the animals had an effect on viral loads in the lung (Supporting Information, Figure S1). Some strains, such as 129S1/SvImJ and CAST/EiJ, had high levels of MA15 RNA on day 4 after infection whereas others, such as NZO/HILtJ and NOD/ShiLtJ, had low levels of MA15 RNA (Figure S2). Strain-dependent variations in viral RNA levels were also observed in PR8-infected mice, although the pattern of high *vs.* low viral RNA levels was not necessarily the same. For example, although NOD/ShiLtJ mice had lower levels of MA15 RNA than did the other mouse strains on day 4, the inverse was true for PR8 RNA levels in this strain (Figure S2). The pattern of viral read-counts for PR8 can be well-explained by *Mx1* alleles in the eight founders (Ferris *et al.* 2013).

Weight loss is a common and quantifiable readout of disease severity in mouse models of IAV and SARS-CoV infection. To assess the effect of genetic background on the severity of IAV and SARS disease, we measured the daily body weights of the mice from time of infection to time of kill. The daily body weights of the mice are provided in Figure S3 for MA15-infected mice and in Figure S4 for PR8-infected mice. A wide variety of phenotypes emerged, ranging from 20% weight loss to essentially no weight loss. Strikingly, NZO/HlLtJ mice, alone among the eight strains, did not lose weight when infected with either PR8 or MA15.

### Thousands of novel intergenic transcripts have potential roles in the host response to IAV and SARS-CoV

The mouse reference annotation is largely based on one founder strain, C57BL/6J, but large genetic variations separate all eight founders, particularly among the three wild-derived strains as well as between the classical laboratory strains and the wild-derived strains (Keane *et al.* 2011). Indiscriminately using the reference annotation in CC transcriptome studies could bias results and lead to inaccurate conclusions. In addition, the advent of RNA-seq has led to large discoveries of novel transcripts (ENCODE Project Consortium *et al.* 2012). Therefore, we built a custom bioinformatics pipeline (Figure S5) for novel transcript discovery. We based our pipeline on the popular novel transcript discovery programs, Cufflinks (Trapnell *et al.* 2012) and Trinity (Grabherr *et al.* 2011), with the former for genome-based transcript discovery and the latter for *de novo* assembly of transcripts not based on genomes. The pipeline accounted for the genetic variations of the founders’ genomes compared with the reference genome, on which the reference annotation is built, by mapping transcripts in the reference annotation to pseudogenomes (Huang *et al.* 2013). The mapped transcripts were used in place of the reference annotation. Because we had total RNA data, we focused on novel intergenic transcripts and filtered novel transcripts by comparing them against known transcripts. Additional filtering steps were required for *de novo* assembled transcripts to exclude false positives, so we mapped them against viral and ribosomal sequences and compared them with all known mouse, human, and rat ESTs and clones using BLAST. We also required all novel transcripts to be at least 200 bp long.

**Figure 1:**
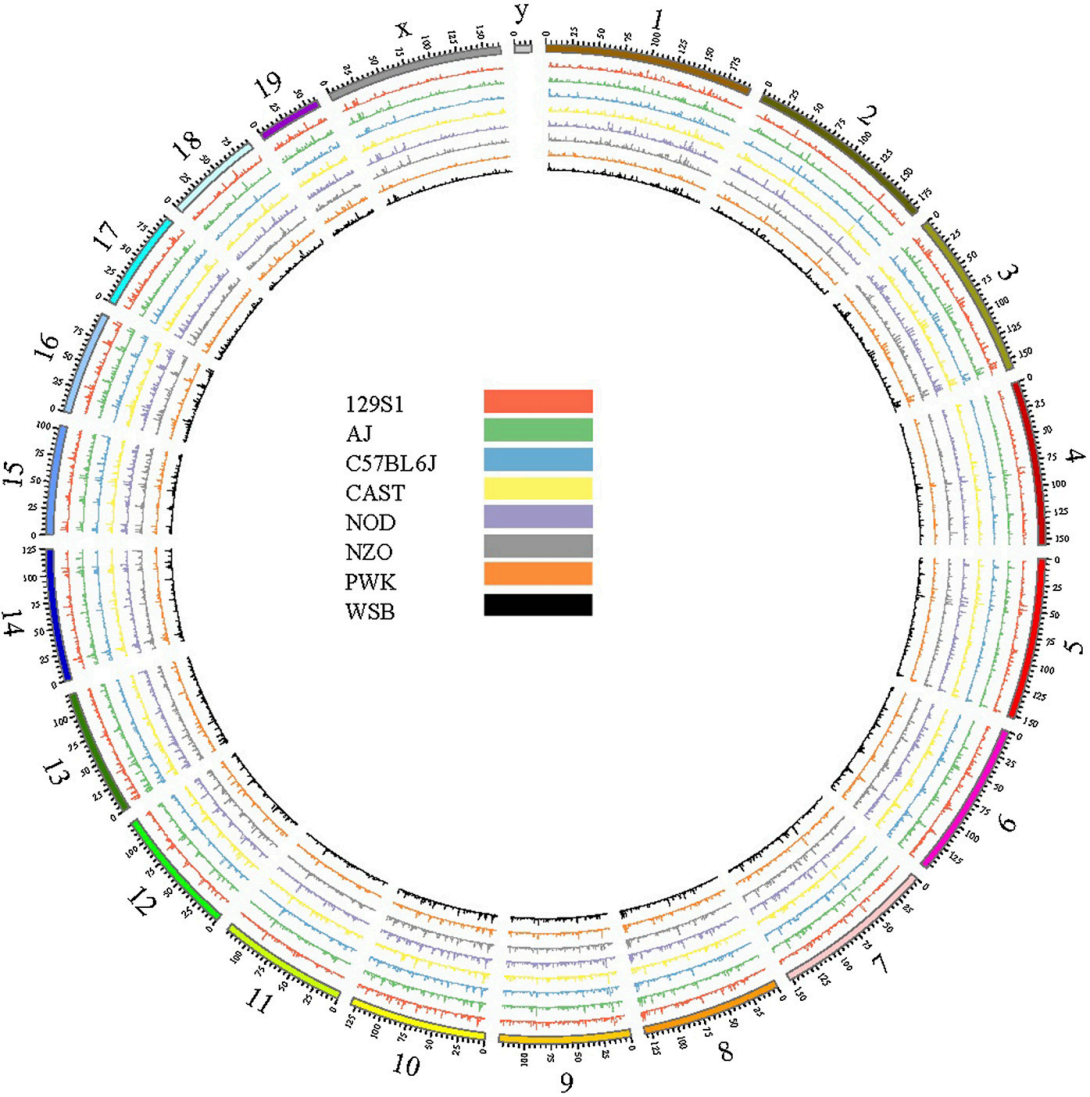
Circos plot of genome-wide novel transcript density. Chromosomes 1 through 19 and X are represented as 20 different segments with their genomic coordinates labeled in megabases. The novel transcript density was defined as the total number of nucleotides within all novel transcripts of a given 100-KB region divided by the total number of nucleotides in that region. We considered imputed introns to be part of novel transcripts to regions of high density often resulting from multiple, overlapping novel transcripts. Because all mice in this report were female, no Y chromosome information is available.

**Figure 2:**
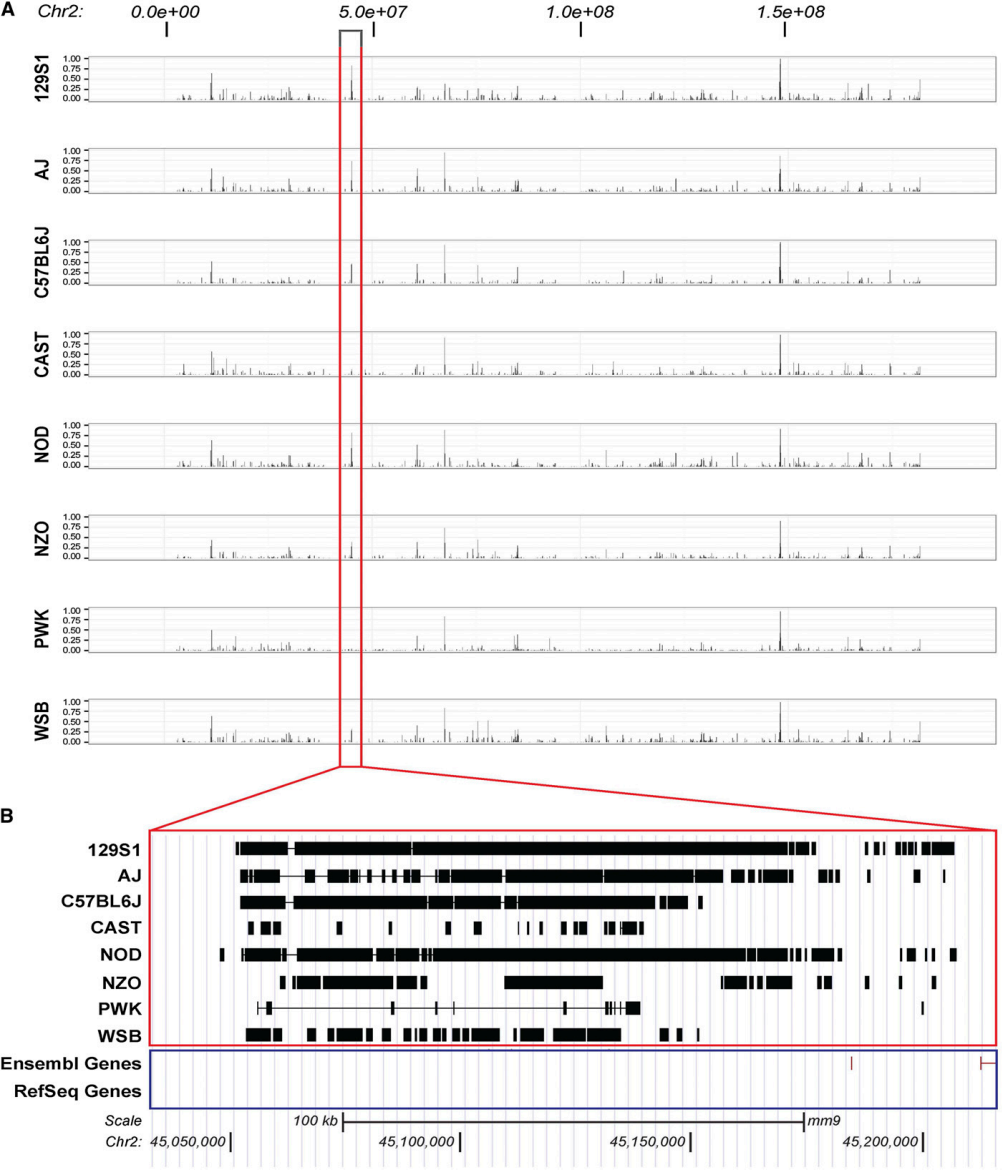
Novel transcript density on chromosome 2. (A) The novel transcript density on chromosome 2 is represented in further detail. The nucleotide position is shown at the top of the figure. Each of the eight sub-panels denotes a single mouse strain. A red box marks a region between 45 Mb and 45.2 Mb where the wild-derived strains, CAST, PWK, and WSB, have shorter or fewer novel transcripts than the other five strains. A magnified view of this region is shown in (B).

The numbers of new intergenic transcripts in all founder strains are shown in Figure S6A. C57BL/6J had the fewest (20887) new transcripts because the reference annotation is based on this strain and therefore is the most complete. We also observed that NOD/ShiLtJ mice had 30695 new transcripts and that all other strains had more new intergenic transcripts than C57BL/6J (Figure S6A). Surprisingly, wild-derived strains did not have more new transcripts than laboratory strains (except C57BL/6J), although overall differences in the numbers of novel transcripts among the other seven strains were not large. This could be due, in part, to the imperfect state of founder pseudogenomes, which are rooted in the reference genome and thus may not accurately describe the genomes of the other seven strains. This is particularly problematic for the wild-derived strains, whose genomes are more divergent from C57BL/6J. When we plotted the density of the novel transcripts onto mouse genomes, we found that although novel transcripts were spread throughout the eight genomes, they did cluster around certain locations (Figure 1). To further investigate this phenomenon, we zoomed-in on chromosome 2 (Figure 2A). The clustering patterns were remarkably consistent between founders, further validating the presence of these novel transcripts (Figure 2A). However, not all locations were identical for the eight founders; some regions showed clear differences between the wild-derived strains and the classical laboratory strains. One such region is found between 45.05 Mb and 45.2 Mb on chromosome 2, where the wild-derived strains tended to have shorter and fewer novel transcripts than the laboratory strains, as illustrated by Figure 2B. This observation led us to cluster the founder strains based on the novel transcript density of chromosome 2. The resulting similarity distance showed that the wild-derived strains were more distant from the laboratory strains, with the WSB/EiJ strain being the most closely related to the laboratory strains (Figure S6B). This observation is similar to that of a previous phylogenetic characterization of inbred mouse strains (Yang *et al.* 2007).

**Figure 3:**
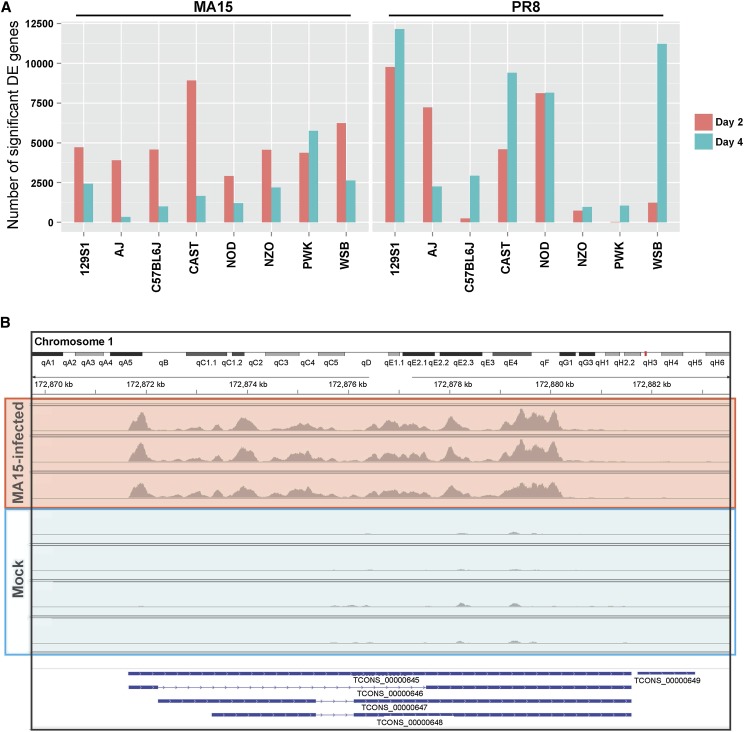
Differential expression of novel transcripts. (A) The number of intergenic novel transcripts that are differentially expressed between infected samples and mocks. Computation was performed using the Bioconductor package “edgeR.” A large proportion of new intergenic transcripts showed differential expression in response to infection, pointing to their potential functional role in the host response. (B) An example of differential expression of novel transcripts in C57BL/6J mice at day 2 after MA15 infection. Note the difference between virus-infected animals (top three samples) and mock-infected animals (bottom four samples). This locus has no annotated gene nearby and is not within known genes, as can be observed by the blank panel of “Refseq genes.”

Because the novel transcripts were induced during viral infection, they may play functional roles in the host response. To gain insight into possible functional roles, we tested for DE genes by comparing samples from virus-infected and mock-infected animals. New intergenic transcripts that were DE in response to infection are summarized in Figure 3A. There were considerably more transcripts in 129S1/SvImJ, WSB/EiJ, CAST/EiJ, and NOD/ShiLtJ mice on day 4 after PR8 infection than on day 4 after MA15 infection, and significantly more new transcripts in 129S1/SvImJ, WSB/EiJ, and CAST/EiJ mice on day 4 after PR8 infection than on day 2 after PR8 infection. The pattern of strain variation with regard to the number of novel DE transcripts was similar to the pattern shown by known DE genes. We present an example of a novel DE transcript in Figure 3B. There were thousands or more reads mapped to this transcript, which was present in C57BL/6J on day 2 after MA15 infection. The differential expression of this transcript was striking, with abundant expression in samples from virus-infected animals and little expression in samples from mock-infected animals.

To further characterize the novel transcripts, we selected novel transcripts within four previously studied QTL regions (Ferris *et al.* 2013) and associated them with annotated genes. A genome browser view of one QTL region, HrI3, is shown in Figure S7. The QTL study used PR8 virus and pre-CC mice, mice that are descendants of the founders in the current study and therefore are particularly relevant. First, we used linear regression to choose novel transcripts associated with weight loss; after multiple-testing correction, we obtained three significant novel transcripts. The three novel transcripts fall into regions previously associated with mouse body weight, pulmonary edema, or airway neutrophils of the mouse response to PR8 and their expression levels have further been associated with weight loss, an indicator of morbidity. Two of these transcripts were found in PWK/PhJ mice and the third transcript was found in C67BL/6J mice. When we used linear regression to characterize the functional roles of the three novel transcripts by associating their expression levels with those of annotated genes, one transcript in PWK/PhJ mice failed to significantly associate with any genes, but the other two transcripts were associated with many. These transcripts included XLOC_000093 in PWK/PhJ mice on chromosome 1 between 21.7 Mb and 29 Mb and XLOC_008559 in C57BL/6J mice on chromosome 15 between 77.4 Mb and 86.6 Mb. By using a cut-off value of 10^−5^ as the threshold, we identified more than 200 annotated genes that associated with these two novel transcripts. When we used IPA functional analysis to associate annotated genes with their predicted biological functions, we found that genes associated with XLOC_000093 were associated with TREM1 signaling, B cell receptor signaling, and NF-κB activation. Given the fact that these novel transcripts are within a QTL region associated with the host response to PR8, are highly associated with weight loss after infection, and are associated with many important immune genes, it is likely that they have a functional role in the host response to viral infection.

### Lack of alignment between *de novo* transcripts and mouse genomes reveals multiple gaps in existing mouse genome sequences

**Figure 4:**
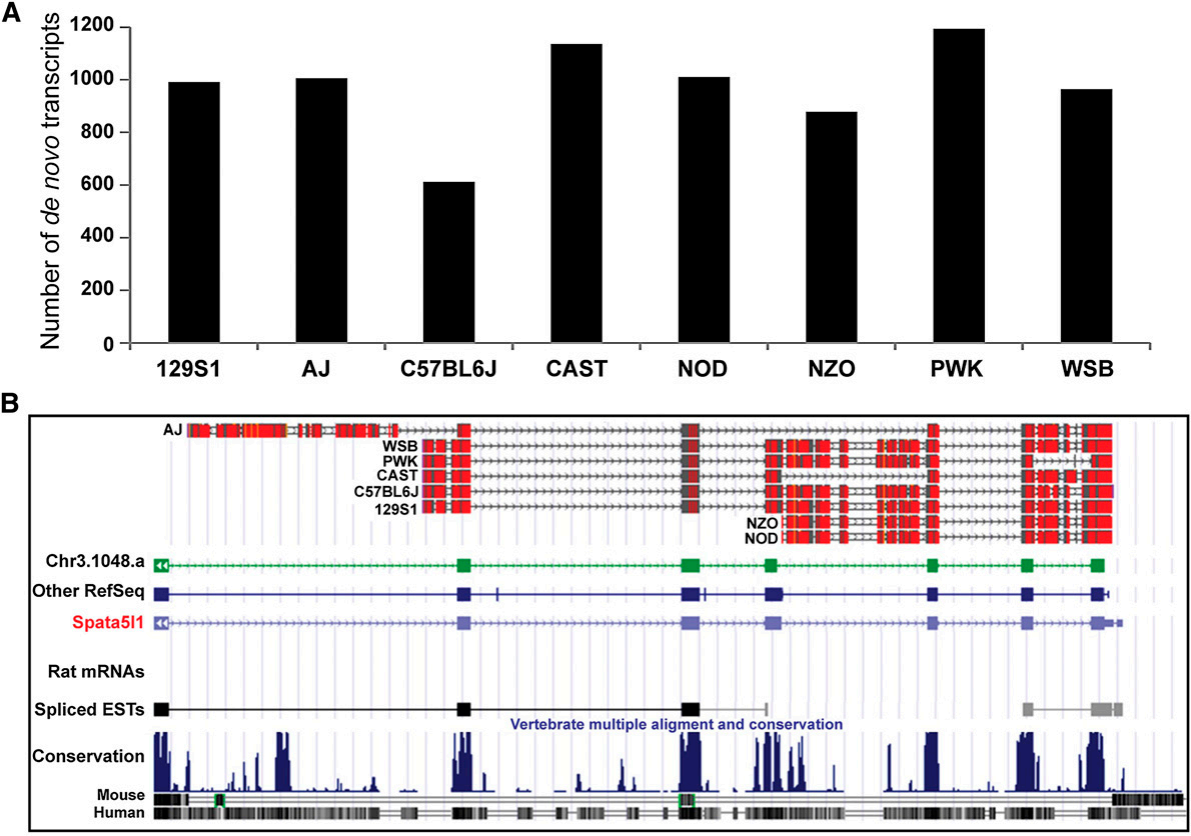
*De novo* transcripts that do not map to existing mouse genes. The *de novo* transcripts were assembled from RNA-seq reads not mappable to pseudogenomes and then the transcripts were filtered by mapping the transcripts to pseudogenomes. The *de novo* transcripts were filtered using BLAST to probe the “nx” database of known mouse, rat, and human sequences. (A) The number of *de novo* transcripts. The *de novo* transcripts were fewest in the C57BL/6J strain, whereas the wild-derived strains, CAST, PWK, and WSB, had more *de novo* transcripts than laboratory strains. (B) An example showing that multiple *de novo* transcripts from all eight founders could be mapped to the human *Spata5l1* gene, spermatogenesis-associated 5-like 1.

Our assembly pipeline not only identified many *de novo* transcripts that did not overlap with known genes but also identified transcripts that could not be placed on either the reference genome or the various founder pseudogenomes. We took a conservative approach to mapping these transcripts to minimize false positives. First, we selected the parts of the output from Trinity that did not map to pseudogenomes or the reference genome. We then filtered the data by first removing transcripts mapped to viral and ribosomal genomes, then by retaining only those transcripts that aligned to known human, rat, and mouse sequences (ESTs or DNA clones), and finally by requiring a minimum length of 200 bp per transcript. A summary of the remaining *de novo* transcripts is presented in Figure 4A. Because both the reference annotation and the reference genome are based on samples from the C57BL/6J strain, it is no surprise that C57BL/6J had the fewest *de novo* transcripts among all the founders. However, it is somewhat surprising that there were ∼600 *de novo* transcripts in this strain. On average, the laboratory strains (A/J, 129S1/SvImJ, C57BL/6J, NOD/ShiLtJ, NZO/HILtJ) had fewer *de novo* transcripts than the wild-derived strains (CAST/EiJ, PWK/PhJ, WSB/EiJ), reflecting the greater genomic difference between the wild-derived strains and C57BL/6J. Some of the *de novo* transcripts were more than 1000 bp long, with the transcripts shown in Figure 4B being prominent examples. These transcripts have very low similarity to the reference genome and pseudogenomes but could be mapped to both rat and human genomes with coverage approximately 70% or more. Interestingly, these transcripts could also be mapped to a lesser known mouse genome assembly called Mm_Celera (Mural *et al.* 2002), which was generated by Celera genomics based on multiple mouse strains. One prominent example is the *de novo* transcripts that spanned multiple conserved exons of the gene *Spata5l1* and aligned well with respective exons of human and rat genomes (Figure 4B). These data provide strong evidence that the existing reference genomes and various pseudogenomes have gaps within well-known coding genes.

To further illustrate the power of our RNA-seq data to uncover gaps in the mouse genome, we recapitulated genomic differences between founders for *Mx1*, an important immune gene. Our groups have shown previously that four of the founders, A/J, C57BL/6J, 129S1/SvImJ, and NOD/ShiLtJ, have a deletion of three exons in the middle of *Mx1*, whereas the other four founders have a full complement of exons (Ferris *et al.* 2013). We compared our *de novo* transcripts that could not be placed on the chromosomes of the pseudogenome with the sequences of exons 9 to 11, the deleted exons. We were able to completely recapitulate the finding of Ferris *et al.* (2013) so that there were transcripts with more than 600-bp overlaps for four founders with full gene models and no match for founders with deleted exons. The alignment of these transcripts to the rat genome showed a similar pattern as observed in Figure 4B, with transcripts showing affinity to conserved exons in the genome of a closely related species (Figure S8).

### Differential isoform expression and the host response to viral infection

The majority of mammalian genes have multiple isoforms (Wang *et al.* 2008; Zhang *et al.* 2013), the significance of which is increasingly being recognized (Trapnell *et al.* 2010). Isoform expression has been linked to the host response to IAV (Terrier *et al.* 2012), and isoforms of immune loci such as HLA have long been recognized as being important to immune function (An *et al.* 2013; Lin *et al.* 2003). RNA-seq is theoretically capable of distinguishing between different isoforms in an unbiased survey, but current short-read technologies make isoform quantification challenging. We circumvented the technical challenges of isoform quantification by focusing on the expression profiles of splice junctions. Because splice junctions are intimately tied to genomic coordinates, we mapped RNA reads to the reference genome and excluded false positives by permitting only canonical splice sites.

The first thing we observed was the distinct expression patterns of alternative splice junctions among the founders (Table S1). We looked for alternative splice junctions expressed in one strain and not the other. A minimum read count of 10 was required for a splice junction to be considered expressed, whereas the maximum read count for a junction to be considered not expressed was five; the gap is a conservative filtering criterion. As expected, there were large strain-specific differences in alternative splice expression with thousands of splice junctions expressed in one founder strain and not the other (Table S1). Wild-derived PWK/PhJ had the highest number of splice junctions expressed that could not be found in other strains. The strain-specific differences in splice junction expression foreshadowed pervasive differences in isoform expression patterns.

Next, we studied differential isoform expression in the context of IAV and SARS-CoV infection. At one extreme, we looked for splice junctions expressed in samples from virus-infected animals but not in samples from mock-infected animals of the same strain (Table S2). Strain differences were clearly evident. The number of splice junctions expressed in samples from virus-infected animals ranged from a few hundred to a few thousand. There were also large differences for individual strains with respect to different viruses and time points.

We also performed a detailed analysis of differential isoform expression for individual genes. Because we were using differential alternative splice junction expression as a proxy for differential isoform expression, we excluded splice junctions inside DE genes to disambiguate real from ambiguous differential alternative splicing due to differential gene expression. Using this approach, we obtained the number of differentially expressed alternative splice junctions (Table S3). Some of them were in known immune genes such as *Irak1* (interleukin-1 receptor-associated kinase 1). The *Irak1* gene only showed differential isoform expression in CAST/EiJ mice, indicating strain-specific expression (Figure S9). The other DE isoforms we discovered also tended to be strain-specific, but this might be due, in part, to our extremely conservative approach to discovering differential isoform expression and the small number of DE isoforms we uncovered.

**Figure 5:**
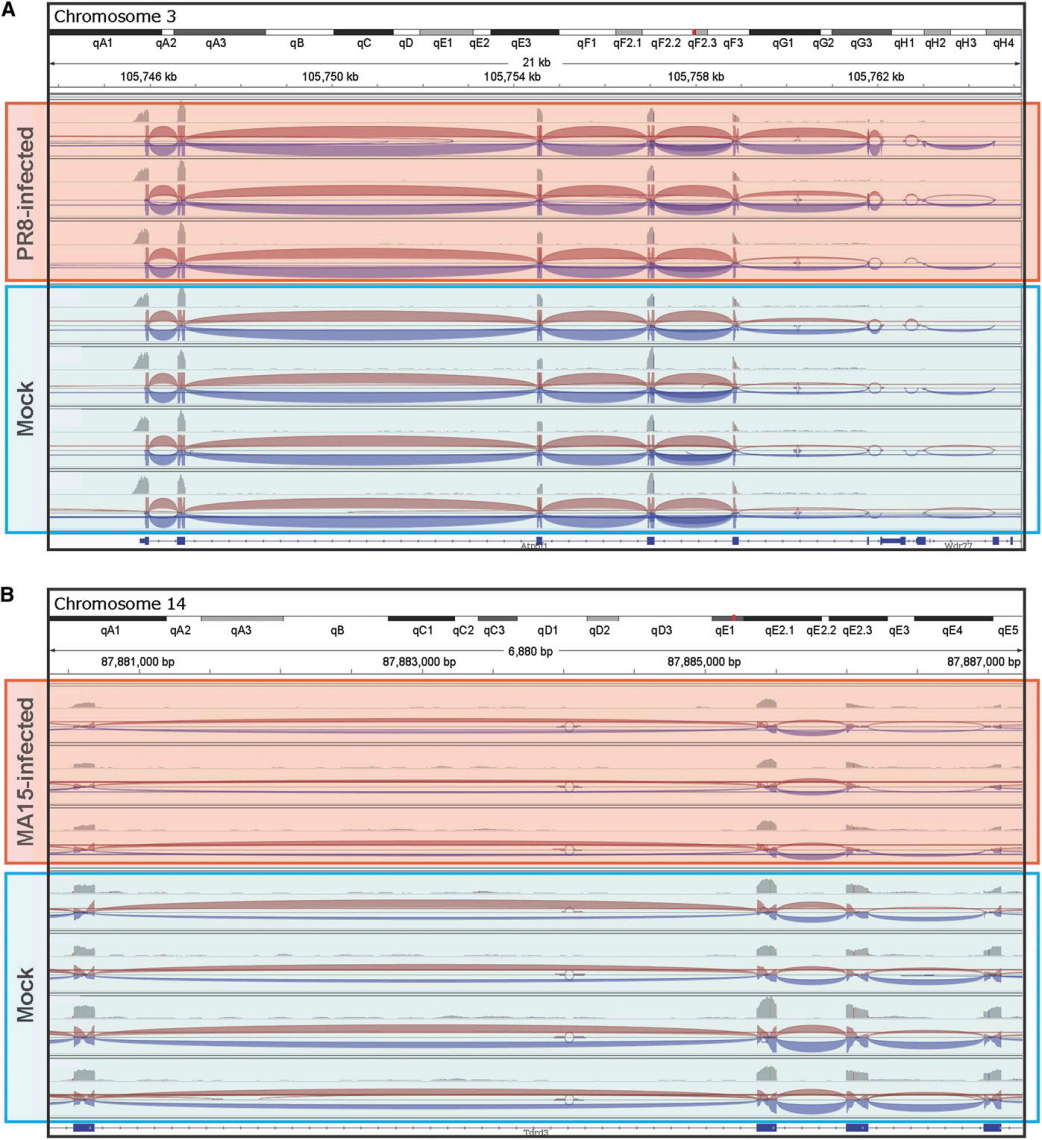
*Cis*-regulated isoform differential expression. Genes *Atp5f1* (A) and *Tdrd3* (B) exhibited *cis*-regulated differential isoform expression in response to infection. Although neither gene showed differential expression overall, they both expressed select splice junctions differentially and contained strain-specific genetic variations within 2 bp of the splice junctions. Gene *Atp5f1* (A) had a splice junction on chromosome 3 from 105758960 to 105761779 that was differentially expressed at day 4 after PR8 infection in WSB mice, which contains a SNP, rs33689100, at 105758959 on chromosome 3 that changes nucleotide C to A. (B) *Tdrd3* is a large gene spanning over 128 kb that exhibits isoform differential expression at day 2 after infection with MA15 in CAST mice. The splice junction on chromosome 14 from 87886156 to 87886968 had substantially fewer read counts in samples from infected animals (top three) than in samples from mock-infected animals (bottom four). The adjusted p-value was 2.93×10^−5^ for the rightmost junction and 0.35 for the entire gene. In addition, Tdrd3 in CAST mice had an insertion at 87886156 bp, which impacted splicing and made the splicing pattern of this strain different from other founder strains.

There are two ways in which genetic variation can influence isoform expression. One is through distant regulation, by changing the expression of transcription factors or their affinity for distant promoters. The other is by changing local sequences such as spliceosome binding sites or intron–exon boundaries. While genetic variation was an obvious source of splicing variations, it was not clear whether differential expression was due to distant regulation or direct sequence alteration of local sites. Distant regulators are difficult to ascertain without a full genetic study, but local sequence variations could be assessed with our dataset. We therefore compared our list of DE splice junctions with published genetic variations obtained through a next-generation sequencing project (Keane *et al.* 2011) and extracted DE isoforms with genetic variations within 2 bp of their intron–exon boundaries. Because of the proximity of these genetic variations to intron–exon boundaries, this subset of DE isoforms is likely the result of *cis*-regulated gene expression. One example is found in the *Atp5f1* gene (Figure 5A). *Atp5f1* is an ATP synthase and transport gene that has been linked to hepatitis B infection (Lee *et al.* 2008). This gene, although not differentially expressed overall, had two splice junctions showing elevated expression in samples from PR8-infected WSB/EiJ mice on day 4 after infection. Furthermore, there was a SNP (rs33689100) at 105758959 on chromosome 3 that was within 2 bp of an intron–exon boundary. Another example was the *Tdrd3* gene, which showed differential isoform expression in CAST/EiJ mice on day 2 after MA15 infection. This gene has been linked to stress granules, which are co-opted by several viruses during replication (Lloyd 2013). At 87886156 on chromosome 14, there was an insertion (rs234605399) within 2 bp of an intron–exon boundary, likely causing the splice junction spanning that position to be differentially expressed (Figure 5B). The splice junction neighboring rs234605399 had far fewer reads in samples from virus-infected animals, although the overall gene expression was similar for all samples.

**Figure 6:**
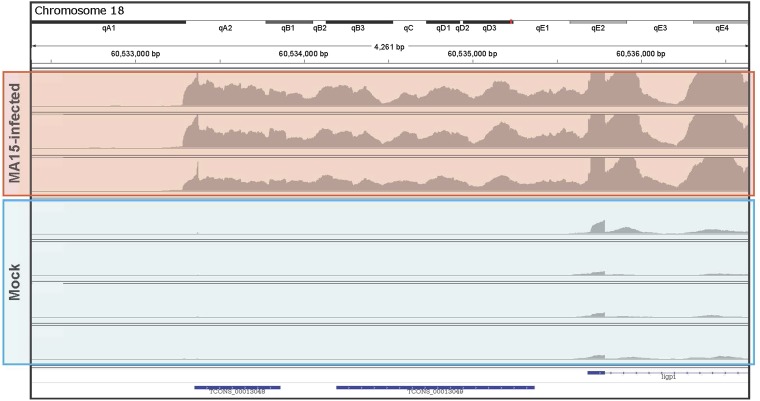
Differential expression of untranslated regions. The 5′ end of the annotated gene *Iigp1* (interferon inducible GTPase 1) contains an unannotated untranslated region (UTR) that is expressed only during infection. The annotated portion was differentially expressed but samples from mock-infected animals had hundreds if not thousands of reads whereas the unannotated 5′ UTR had practically none. This was observed in C57BL/6J mice, suggesting that the *Iigp1* gene model is incomplete in the reference annotation.

We also identified unannotated untranslated regions (UTRs) in our results, although we did not systematically search for unannotated UTRs. *Iigp1* (interferon-inducible GTPase 1) has an unannotated 5′ UTR that is highly expressed in samples from infected animals (Figure 6). This is an example of differential isoform expression with different starting sites. Although the functional roles of the unannotated 5′ UTR and differential isoform expression are not clear, given the role that *Iigp1* has in modulating the interferon-γ (Boehm *et al.* 1998) response, it is possible that its 5′ UTR plays a role in driving the host response.

### Genome sequence variations and tight regulation drive strain-specific gene expression

**Figure 7:**
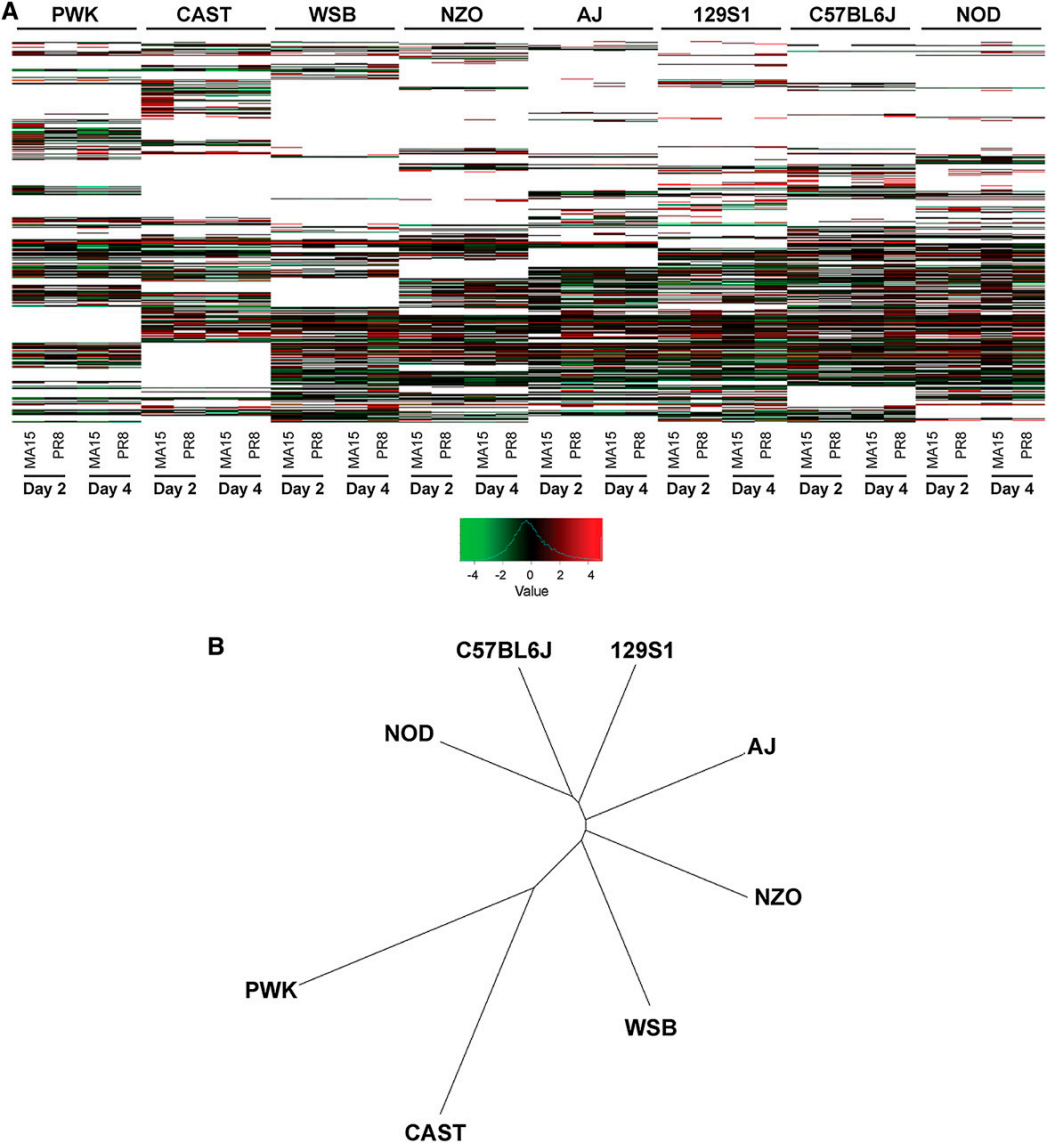
Some differentially expressed genes are not expressed in one or more founder strains. Of the 9149 DE genes, 2150 lacked expression in one or more founder strains. (A) Log-fold changes are shown as a heat map. The pattern of white space, which denotes nonexpression, clearly discriminates the founder strains. (B) Clustering the strains based on nonexpression pattern resulted in a graph resembling a previous phylogenetic tree computed from genetic variations of the founder strains (Yang *et al.* 2007).

We performed differential expression analysis on annotated genes and found widespread strain specificity in the numbers and the functional enrichment of DE genes; the details of which can be found in the Supplementary Materials in File S1. Although it was clear that genetic differences drove the strain-specific expression patterns that were described above, it was unclear whether this was by indirect genetic regulation or by direct genome sequence changes. Examples of direct sequence alteration include premature transcriptional stops and reduced transcription factor–binding affinity. To tease apart these sources of distinct expression patterns and their likely functional roles, we first separated the DE genes into three groups: those not expressed in all the samples of one mouse strain (group A); those expressed in all strains (group B); and the remainder. In other words, group A genes were differentially expressed during virus infection for at least one strain but were not expressed for another strain for all time points and all viruses (Figure 7A). This group includes genes with extreme expression difference between strains. We used group A gene expression levels to cluster all founder strains and discovered that the resulting similarity graph (Figure 7B) resembled the phylogenetic tree based on genetic variations (Yang *et al.* 2007). Using the remaining DE genes, or all DE genes, did not yield the same similarity graph. This suggests that genome sequence variations between founders had a large and immediate impact on the expression of these genes. The genome sequence variation driving selective expression of these genes is likely nearby in upstream binding sites or promoters, which are different between strains because of SNPs, small indels, or structural variations. In extreme cases, whole genes may be deleted in certain strains. Although genetic regulation is always a factor in gene expression, for these genes, expression differences between founder strains may be more directly explained by genome sequence variations within or near those genes.

**Figure 8:**
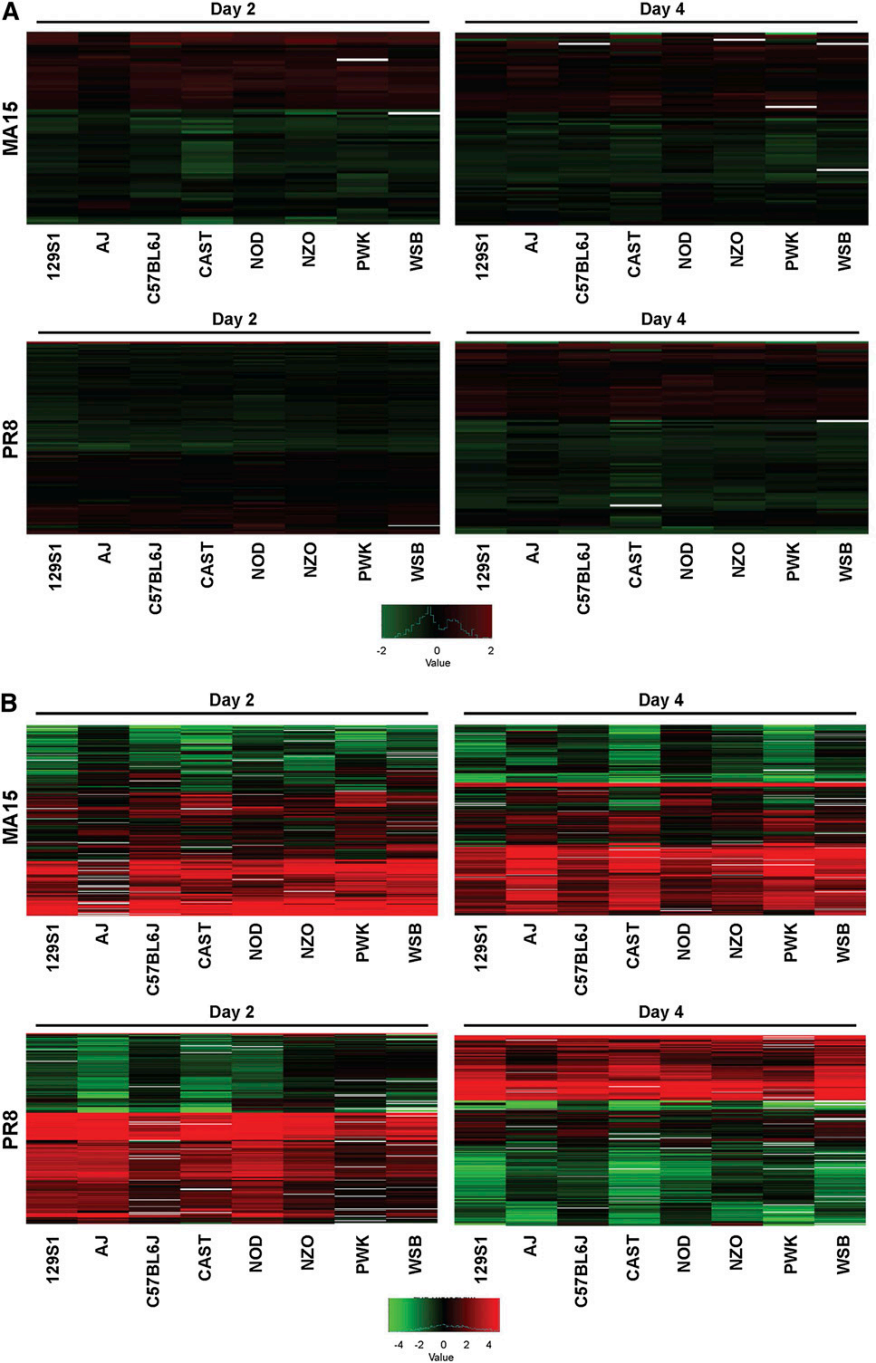
Highly variably and very stably expressed DE genes. Of the genes that were differentially expressed in all strains between infected and mock-infected animals, 445 were consistently expressed whereas 836 were highly variably expressed. Their respective log-fold change patterns are shown in (A) and (B), respectively. The functional enrichments of the respective genes are shown in Table 1 and Table 2.

We further divided the DE genes belonging to group B into two categories using median absolute deviation (MAD) of log-fold change as the metric. If a gene had a MAD score less than 0.1 within a unique combination of time point and virus, then it was considered a consistently expressed gene (Figure 8A); if the MAD score was more than 1, then it was considered a gene with highly variable expression (Figure 8B). Consistently expressed genes included genes that were similarly expressed across all eight founders, whereas genes with highly variable expression between strains are likely to be ultimately responsible for differing phenotypes. This is a further gradation of genes with strain-specific expression patterns; the highly variable genes have strain-specific pattern, but the stably expressed genes do not. Interestingly, of four combinations, PR8 *vs.* MA15 at day 2 or day 4 after infection, consistently expressed genes did not overlap among different viruses and time points, and neither did genes with highly variable expression (not shown). This suggests a tightly controlled host-response program in response to infection. The expression patterns shown in Figure 8, A and B have 445 consistently expressed genes and 835 genes with highly variable expression. When we looked for pathway enrichment using GO terms, we found the consistently expressed genes were dominated by general biological processes and cellular functions, with low enrichment of immune functions or pathways. The top 10 enriched pathways for consistently expressed genes are in Table 1. There, only one pathway, pro-B-cell differentiation biological process, was related to immune function. The opposite was true for genes with highly variable expression, whose GO terms were predominantly immune functions such as innate immune system and interferon stimulation. The top 10 enriched pathways are listed in Table 2. Functional enrichment of immune functions remained high regardless of whether all known genes or just the DE genes were used as the background, so the high functional enrichment of genes with highly variable expression was not confounded by the enrichment of DE genes in immune functions. It is possible that consistently expressed genes are largely responsible for maintaining basic biological functions, whereas genes with highly variable expression between strains are responsible for the manifestation of different host responses.

**Table 1:** Functional enrichment of consistently expressed genes

**Protein stabilization**	**0.00054**
**Biological process**	**0.0011**
**Positive regulation of phosphoprotein phosphatase activity**	**0.0012**
**Cerebral cortex radially oriented cell migration**	**0.0017**
**Bicarbonate transport**	**0.0024**
**Positive regulation of endothelial cell migration**	**0.0026**
**Pro-B cell differentiation**	**0.0027**
**Sesquiterpenoid metabolic process**	**0.0027**
**Pyrimidine nucleoside monophosphate biosynthetic process**	**0.0027**
**Chaperone cofactor-dependent protein refolding**	**0.0027**

The top 10 enriched GO terms for genes consistently stably expressed among all founder strains. The adjusted p-values are listed to the right. A distinct lack of immune-related enrichment can be observed for stably DE genes derived from comparing infected mice with mock-infected mice.

**Table 2:** Functional enrichment of highly variably expressed genes.

**Cellular response to interferon-gamma**	**2.41E−09**
**Cellular response to interferon-beta**	**2.46E−07**
**Cellular response to lipopolysaccharide**	**5.20E−07**
**Innate immune response**	**2.21E−06**
**Positive regulation of angiogenesis**	**7.20E−06**
**Defense response to protozoan**	**8.26E−06**
**Regulation of the force of heart contraction**	**1.57E−05**
**Neutrophil chemotaxis**	**1.59E−05**
**Immune response**	**1.72E−05**
**Brown fat cell differentiation**	**3.25E−05**

The top 10 enriched GO terms for genes variably expressed among all founder strains. The adjusted p-values are listed to the right. Dominance of immune-related functions can be seen in enrichment whose baseline is all DE genes, which already has high enrichment for immune functions

### Widespread strain-specific gene expression points to diverse host responses to viral infection

We mapped short RNA-seq reads to pseudogenomes and generated read counts based on the reference annotation adapted to pseudogenomes to obtain expression profiles for all 124 samples. Five samples were excluded from the gene expression analysis due to unusually low viral read counts and expression similar to that of the mock samples. The number of replicates for each comparison is listed in Table S4. The remaining samples showed a clear separation of infected and mock samples, which is apparent in an MDS plot for all samples (Figure S10). For samples from A/J strain day 2 after being infected with MA15, the MDS plot shows clear separation between infected samples and mock samples (Figure S11). Differential expression analysis comparing infected samples against mock samples within each founder strain was performed using edgeR (Robinson *et al.* 2009) with a stringent criterion of FDR <0.01 and a fold change of ±2.

There were considerable strain-specific differences for both PR8 and MA15 on days 2 and 4 after infection. These were apparent even at the summary level of the numbers of differentially expressed genes (Figure S12). For example, PWK/PhJ had far fewer DE genes (223) than the other strains on day 2 after PR8 infection, whereas NOD/ShiLtJ had the most DE genes (2454). At day 4 after infection, all PR8-infected strains had markedly increased numbers of DE genes, except for A/J, which had a substantial decrease (from 1868 to 702). However, MA15-infected animals had fewer DE genes on day 4 than on day 2 or similar numbers of DE genes on both days. The largest number of DE genes (3748 in PR8-infected 129S1/SvImJ on day 4) was approximately an order of magnitude greater than the smallest number of DE genes (223 in PR8-infected PWK/PhJ on day 2), indicating that genetic variation among the founders was driving differences in the host response to viral infection.

Strain variation in the number of DE genes was reflected in functional analysis. When IPA was used to identify pathways that were enriched in DE genes, key immune pathways such as granulocyte adhesion and diapedesis, agranulocyte adhesion and diapedesis, crosstalk between dendritic cells and natural killer cells, and TREM1 signaling were among the top 10 enriched pathways with enrichment scores (-log p-value) ranging from more than 3 to more than 20. Strain differences are profound for individual pathways. One such pathway was “granulocyte adhesion and diapedesis,” which is a key innate immune pathway related to vascular cuffing. As shown in a radial plot of the enrichment scores of all eight founders, PR8-infected PWK/PhJ mice had the lowest enrichment score (4.34) on day 2 after infection, whereas NZO/HILtJ had the highest enrichment score (22.67) (Figure S13).

Select genes important to immune response were largely responsible for the vastly different enrichment scores for pathways such as “interferon signaling.” Two examples were interferon-γ and IL-10. Interferon-γ has been shown to protect mice from influenza virus if administrated at an early stage of infection (Sainz *et al.* 2004; Weiss *et al.* 2010), and genetic variation within the *IFNG* gene has been linked to SARS in humans (Chong *et al.* 2006). IL-10 is an anti-inflammatory cytokine that is linked to influenza recovery (McKinstry *et al.* 2009) and the effectiveness of SARS treatment (Ng *et al.* 2004). Its expression can also predict patient survival among people infected with emergent H7N9 virus (Wang *et al.* 2013). Given their pivotal role in fighting IAV and SARS, it was intriguing that these genes showed strain-specific differential expression. Among mice infected with PR8 at day 2 after infection, *IFNG* was only expressed in C57BL/6J, CAST/EiJ, PWK/PhJ, and WSB/EiJ, whereas it was expressed in all strains except CAST/EiJ on day 4 after infection. A recent article also reported an absence of *IFNG* expression in the lungs of CAST/EiJ mice (Earl *et al.* 2012). For SARS-CoV infection, *IFNG* was expressed in all but the CAST/EiJ strain at day 2 and in all strains at day 4.

### Viral-read percentages are highly predictive of weight loss

We separated short reads mapped to ribosomes and viruses from those mapped to mouse genomes. The numbers of short reads mapped to host, ribosomes, and viruses are shown in Figure S1. To better compare viral reads, viral read counts were normalized to host read counts, which varied as a result of sequencing technology and our sequencing depth requirement, to yield viral read percentages (Figure S2). Viral read counts of mocks are in Table S5.

The number of viral reads and viral titer both measure viral concentration and could be related and to some extent can gauge the extent of viral replication. Total viral read counts and viral read percentages varied greatly among founders, and with different viruses and at different time points (Figure S1 and Figure S2). In particular, the CAST/EiJ strain had far more viral reads than did the other strains on day 2 after MA15 virus infection, especially compared to A/J strain, which had 11-fold fewer viral read counts; this further elaborated earlier observations about the CAST/EiJ strain among four founders (Peng *et al.* 2010). Clear but less extreme variations could be observed in other strains.

Mouse weights were monitored daily because weight loss is considered a clinical sign of morbidity caused by viral infection. All mice except those killed at day 2 had body weights monitored up to day 4 after infection. The weight loss curves, defined as the percentage difference between day 0 weights and subsequent days’ weights, are shown in Figure S3 for MA15 samples and in Figure S4 for PR8 samples. In general, the NZO/HILtJ strain showed little weight loss for both viruses and on all days. NOD/ShiLtJ, A/J, and C57BL/6J strains recovered some weight loss on day 4 after MA15 infection, but no strains showed similar weight recovery with PR8 infection. CAST/EiJ mice lost substantial weight by day 4, particularly when infected with MA15, with weight loss of more than 20% being observed. Interestingly, the CAST/EiJ strain had high viral read counts on day 2 after MA15 infection that decreased substantially by day 4, whereas the mice continued to lose almost 10% of their weight.

Previous reports have shown that viral titer could predict weight loss (Ferris *et al.* 2013). We also found that there was a strong correlation between viral-read percentages and day 4 body weight loss (Spearman correlation coefficient = −0.73) and that viral-read percentages were strongly predictive of weight loss at day 4 (p-value <1.34×10^−10^). There was no correlation between viral-read percentages and day 2 weight loss, probably because it was too early in the course of infection for body weight to be impacted by infection. The scatter plot of viral-read percentages and day 4 weight loss is shown in Figure S14, in which a clear linear relation can be observed.

## Discussion

In this study, we characterized the transcriptional landscape of the eight CC founder strains in response to respiratory viral infection and identified thousands of new transcriptional elements yet to be recorded in the mouse reference annotation. The transcriptional patterns were starkly different between the eight founders, mirroring the phenotypic diversity observed in our study as well as other studies. We observed extreme phenotypes in weight loss and survival among the CC founder strains, similar to that reported in previous studies of mouse genetic regulation of IAV pathogenesis (Ferris *et al.* 2013) and immune cell associations (Phillippi *et al.* 2014) using incipient CC lines. The diversity of phenotypes observed in these studies mimics the diverse clinical symptoms of patients infected with either IAV or SARS-CoV (Pabst *et al.* 2011; Igusa *et al.* 2012; Zaas *et al.* 2009).

As more quantitative trait loci (QTL) studies with CC mice (Durrant *et al.* 2011; Zheng *et al.* 2012; Flint and Eskin 2012) are undertaken, the need for a complete reference genome and annotation will increase. Even though C57BL/6J is the reference strain, we nevertheless found hundreds of missing sequences and tens of thousands of intergenic transcripts in this strain. The other seven strains had even more novel features. It is likely that many of the intergenic transcripts were the extended fragments of known genes, or that multiple new transcripts came from one new gene, so that the total numbers of novel transcripts are lower than estimated. However, the existence of large numbers of unannotated or extended genes is undoubtedly real in the mouse as well as other species. The implication of incomplete reference genome and annotation for QTL and eQTL studies is substantial. Although the transcriptome data presented here will help overcome these shortcomings in mouse studies, the concern remains for other systems, such as human GWAS. The potential pitfalls of incomplete genomes were illustrated by the example of *Spata5l1*, which had multiple exons missing in all eight strains. For studies of genetic control of transcription, our results can help by pointing out potentially incomplete gene models in the reference annotation and can also help complete the mouse annotation with new intergenic transcripts. Our results have particular relevance for viral infection studies, particularly respiratory viruses, as these new transcripts in lung may have functional roles, as evidenced by their differential expression in response to infection.

We also observed that genes showing strain-specific differential expression patterns were heavily enriched in immune pathways, while genes showing generic differential expression patterns had more generic biological functions. This phenomenon may be due, in part, to immune cell infiltration. It is possible that the genes with strain-specific expression are genes that are expressed by different infiltrating cell populations, which may vary between the founders, whereas the nonstrain-specific genes are not active in infiltrating cells. An alternative explanation is that gene expression changes that were not strain-specific are so fundamentally important to mouse survival that they are similarly regulated in all founders. Further study of a variety of CC lines and/or by cell sorting and cell-specific transcriptional analysis could help to explain this phenomenon and its implications on host survival.

Our results may also be used to prioritize candidate genes identified by QTL analysis. For example, the 1700026D08Rik gene is associated with day 4 weight loss after PR8 infection (Ferris *et al.* 2013). We found that the same gene had DE isoforms in NOD/ShiLtJ mice at day 2 after PR8 infection. Although there was no genetic variation near intron–exon boundaries of this gene in NOD/ShiLtJ mice, this strain-specific isoform expression pointed to possible genetic influence and therefore higher likelihood of being the causal gene among the 69 genes and 10 noncoding RNAs that were significantly associated with day 4 weight loss along with 1700026D08Rik. This is especially important because many genes, such as 1700026D08Rik, have poorly described or no known functions. We found that genes such as *IL6*, *Sh3gl3*, and *Atxn10*, which had previously been associated with different phenotypic outcomes in response to IAV infection, also expressed different isoforms in response to SARS-CoV infection. In this case, they have not been identified as QTL candidate genes for SARS-CoV infection, but their likely importance in the host response is increased because their genetic variations may cause large phenotypic variations and their isoforms were differentially expressed in samples from SARS-CoV–infected animals. Because CC mice still have relatively large recombination intervals, the resolution of QTL using CC mice is not at the gene level, and QTL often will have many candidate genes within a significant region. Isoform differential expression results could prove very helpful in finding causal genes in future QTL studies using CC mice by searching for strain-specific DE isoforms among candidate genes. Candidate genes whose protective alleles differentially express isoforms and whose deleterious alleles do not differentially express isoforms are likely the causal genes.

This study is a first important step in extracting full information from transcripts in CC mice. We have provided examples and catalogs of discrepancies with the reference genome and reference annotation. Our differential expression analysis and subsequent functional analysis revealed distinct and focused host responses to viral infection across the eight CC founder strains. The full extent and importance of isoform differential expression are currently unclear. However, it is clear that genetic diversity drives diverse, intricate, and multi-faceted immune responses at the isoform, gene, and pathway levels through direct alteration of gene sequences, indirect regulation of expression, or a combination of both. The transcriptome data from this study are a resource to the mouse genetics community and are of direct benefit to researchers studying transcriptional complexity in genetically diverse populations.

Our study is but one step toward characterizing the transcriptome of genetically diverse mice. mRNA-seq data and Cap analysis gene expression (CAGE) data in addition to our total RNA-seq data can improve gene models for annotated genes and uncover alternative transcription start and end sites. A dearth of good, full genomes for the founders hampered our comparison across strains. Relative small sample sizes for unique combinations of strain, virus, and days after infection are problematic. Considerable variability in mouse body weight within one strain has been observed, but there were too few mice to properly estimate true variance. Future studies can solve these issues by leveraging our data and results.

## Supplementary Material

Supporting Information
