# Supplementary material for: Genomic Profiling of Collaborative Cross Founder Mice Infected with Respiratory Viruses Reveals Novel Transcripts and Infection-Related Strain-Specific Gene and Isoform Expression
Source: G3 (Bethesda). 2014 Jun 5;4(8):1429–44. doi: 10.1534/g3.114.011759 (PMC4132174; doi:10.1534/g3.114.011759)
Supplement: Supporting Information [file supp_4_8_1429__index.html]

Genomic Profiling of Collaborative Cross Founder Mice Infected with Respiratory Viruses Reveals Novel Transcripts and Infection-Related Strain-Specific Gene and Isoform Expression — Supporting Information 

# Genomic Profiling of Collaborative Cross Founder Mice Infected with Respiratory Viruses Reveals Novel Transcripts and Infection-Related Strain-Specific Gene and Isoform Expression

## Supporting Information for Xiong *et al.*, 2014

**Files in this Data Supplement:**

- Supporting Information - File S1, Figures S1-S14, and Tables S1-S5 (PDF, 1 MB)
- File S1 - Transcript discovery pipeline. (PDF, 113 KB)
- Figure S1 - The number of short RNA reads that map to host, ribosomes, and viruses. (PDF, 370 KB)
- Figure S2 - The percentage of RNA reads that belong to MA15 or PR8 genomes. (PDF, 358 KB)
- Figure S3 - Weight loss of founder mice infected with MA15. (PDF, 479 KB)
- Figure S4 - Weight loss of founder mice infected with PR8. (PDF, 479 KB)
- Figure S5 - Schematic of the annotation pipeline. (PDF, 337 KB)
- Figure S6 - Summary of novel transcripts in eight founder strains and their relationships. (PDF, 319 KB)
- Figure S7 - An IGV view of annotated and novel transcripts within HrI3 (chr1:21,767,867–29,085,401) which was found to be associated with pulmonary edema in mice infected with PR8 (Ferris et al. 2013). (PDF, 359 KB)
- Figure S8 - Alignment of de novo transcript with affinity to four extra *Mx1* exons. (PDF, 749 KB)
- Figure S9 - Differential isoform expression of Irak1 gene in MA15-infected CAST mice at day 2 post- infection by MA15. (PDF, 441 KB)
- Figure S10 - MDS plot of all 119 lung samples that were analyzed for differential expression. (PDF, 384 KB)
- Figure S11 - The MDS plot of six samples for AJ strain infected with MA15 at day 2 post infection. (PDF, 341 KB)
- Figure S12 - Differentially expressed genes among the eight founder mouse strains following either influenza or SARS-CoV infection. (PDF, 357 KB)
- Figure S13 - Radial plot showing functional enrichment of granulocyte adhesion and diapedesis pathway genes. (PDF, 308 KB)
- Figure S14 - Percent weight loss correlates with viral read percentages. (PDF, 358 KB)
- Table S1 - The number of splice junctions that were discovered in one strain but not in another strain. (PDF, 112 KB)
- Table S2 - The number of splice junctions that are observed in infected samples but not in mock samples. (PDF, 111 KB)
- Table S3 - The number of differentially expressed splicing junctions. (PDF, 112 KB)
- Table S4 - The replicate size for each combination of mouse strain, virus, and days-post-infection. (PDF, 117 KB)
- Table S5 - Viral read counts in the mock-infected founders. (PDF, 112 KB)
