# Supplementary material for: Genomic Profiling of Collaborative Cross Founder Mice Infected with Respiratory Viruses Reveals Novel Transcripts and Infection-Related Strain-Specific Gene and Isoform Expression
Source: G3 (Bethesda). 2014 Jun 5;4(8):1429–44. doi: 10.1534/g3.114.011759 (PMC4132174; doi:10.1534/g3.114.011759)
Supplement: Supporting Information [file supp_g3.114.011759_011759SI.pdf]

G3 · Genes | Genomes | Genetics

Genomic profiling of Collaborative Cross founder mice infected with respiratory viruses reveals novel transcripts and infection related strain-specific gene and isoform expression

Hao Xiong[*,†], Juliet Morrison[*,†], Martin T. Ferris[†,§], Lisa E. Gralinski[†,‡], Alan C. Whitmore[†,§], Richard Green[*,†], Matthew J. Thomas[*,†], Jennifer Tisoncik-Go[*,†], Gary P. Schroth[**], Fernando Pardo-Manuel de Villena[§], Ralph S. Baric[†,‡], Mark T. Heise[†,§], Xinxia Peng[*,†], and Michael G. Katze[*,†,1]

[*]Department of Microbiology, School of Medicine, University of Washington, Seattle, Washington, United States of America
[†]Pacific Northwest Regional Center of Excellence for Biodefense and Emerging Infectious Diseases Research, Portland, Oregon, United States of America
[‡]Department of Epidemiology, University of North Carolina-Chapel Hill, Chapel Hill, North Carolina, United States of America
[§]Department of Genetics, University of North Carolina-Chapel Hill, Chapel Hill, North Carolina, United States of America
[**]Illumina, Inc., San Diego, California, United States of America
[1] To whom correspondence should be addressed.

Data Access: GSE52405

DOI: 10.1534/g3.114.011759

File S1

Transcript discovery pipeline

The transcript discovery pipeline accomplishes two tasks, adapting existing gene models to the different founder genomes and uncovering new transcripts not overlapping with any known transcripts. To use the most accurate genome for each founders we downloaded the pseudogenomes that had been generated by University of North Carolina based on DNA-seq data produced by the Sanger Institute. This incorporates single-nucleotide variations (SNV), indels, and other structural variations, which can number in the millions, and represents our most current description of the founder genomes. The reference annotation describes over thirty thousand genes, of which about twenty four thousand genes are coding genes. We mapped short reads to their respective pseudogenomes and used the Cufflinks program to predict new transcripts. To avoid confounding intronic reads, we retained only intergenic transcripts. We also took unmapped reads and used Trinity to discover *de novo* transcripts. After filtering *de novo* transcripts that bore resemblance to viral or host genomes, we combined the Trinity output that mapped to mouse genomes with the Cufflinks output, while the stand alone *de novo* transcripts were further filtered to identifythose that were similar to known mouse, rat, or human sequences. The outline of our novel transcript discovery pipeline built is shown in Figure S5.

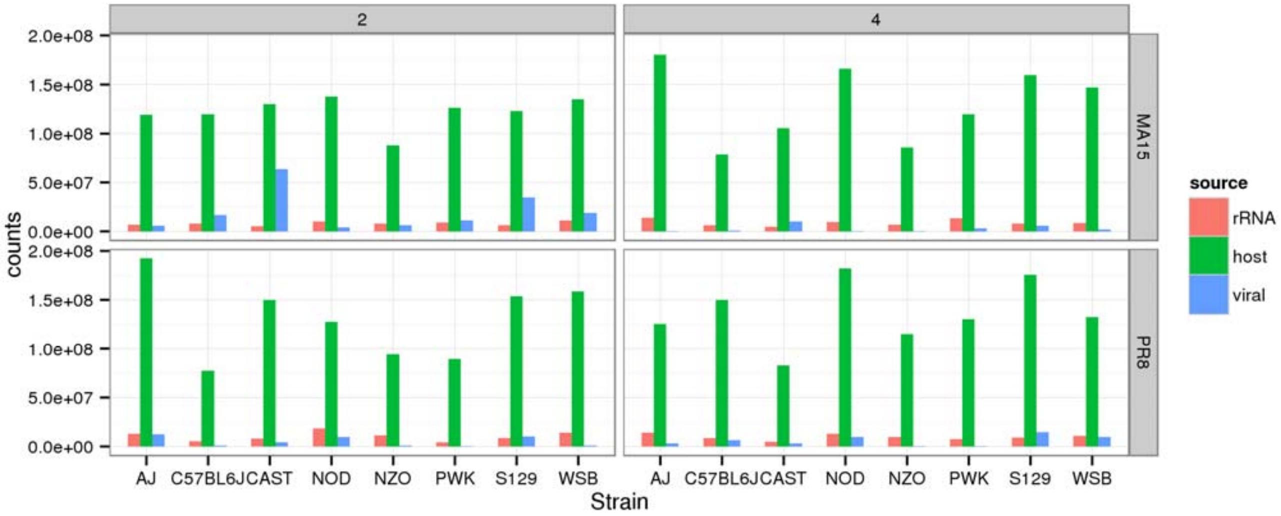

Figure S1 The number of short RNA reads that map to host, ribosomes, and viruses. The read counts were summed by the unique combination of mouse strain, virus, and day post infection. We generally ensured each sample has at least 30 million host reads. The varying sequencing depth for ribosomal and host RNA among strains resulted from random sequencing variation. On the other hand, the viral read counts exhibited strain specificity. For example, MA15-infected CAST mice had 60M viral reads on day 2, while MA15-infected NZO mice had about 6M viral reads, which pointed to strain differences in viral replication.

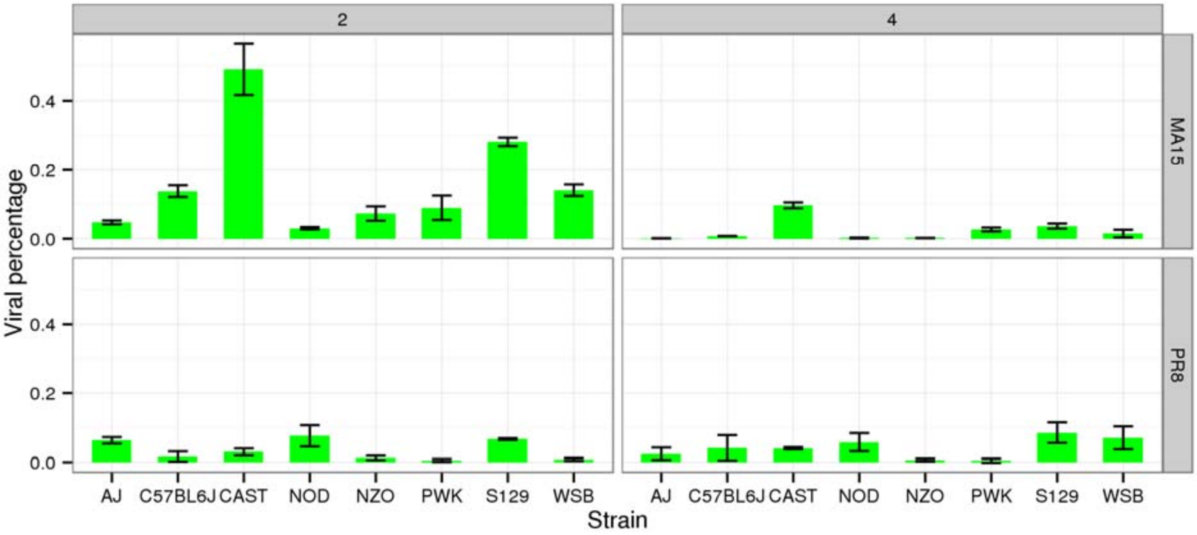

Figure S2 The percentage of RNA reads that belong to MA15 or PR8 genomes. The percentages were calculated by summing all viral reads from animals infected with one virus type for each founder strain at different time points (2 and 4 days post infection) and divided by the total read counts. Each strain had at least two replicates and most have three replicates. There are clear strain-specific differences in the viral read percentages, ranging from almost zero percent of RNA reads in MA15-infected AJ mice to close to half of reads in MA15-infected CAST mice on day 2 post infection.

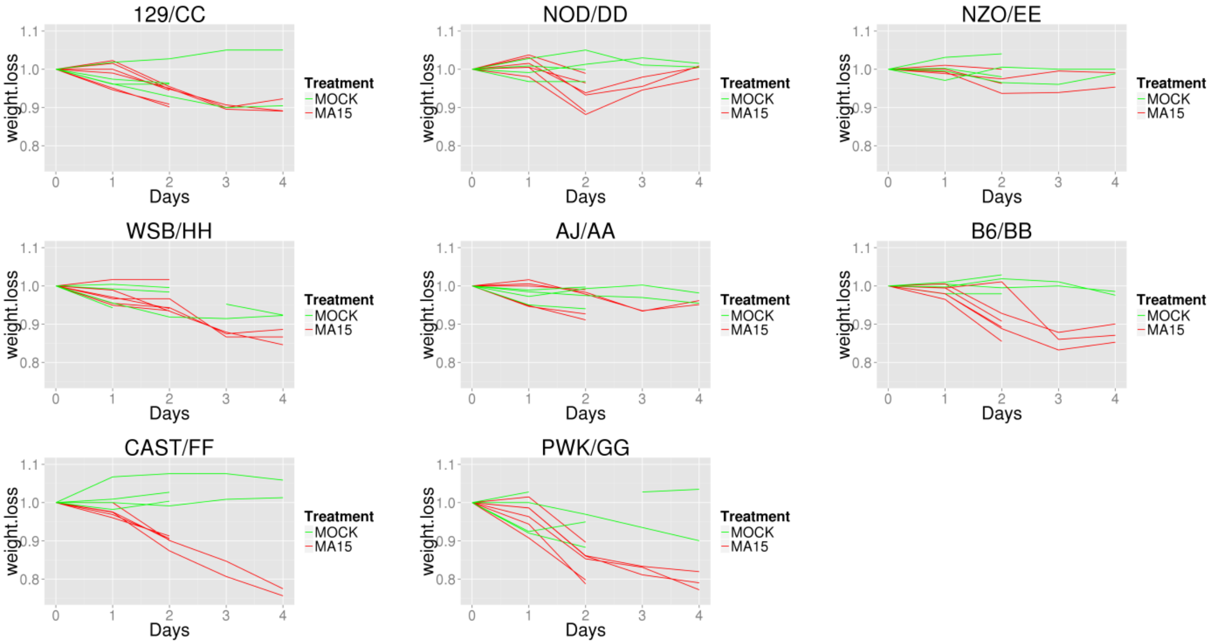

Figure S3 Weight loss of founder mice infected with MA15. Mice were infected with 10^5 PFU of MA15 and monitored for weight loss for 4 days. Half of the infected animals were sacrificed for expression profiles at day 2 post- nfection, while the second half were sacrificed at day 4 post infection. In general, there were observable strain differences in weight loss. For example, NZO lost less than 5% weight, while NOD and AJ mice were able to regain their lost weight by day 4. In contrast, the remaining mouse strains sustained about 10% weight loss, with the CAST and PWK strains showing close to 20% weight loss.

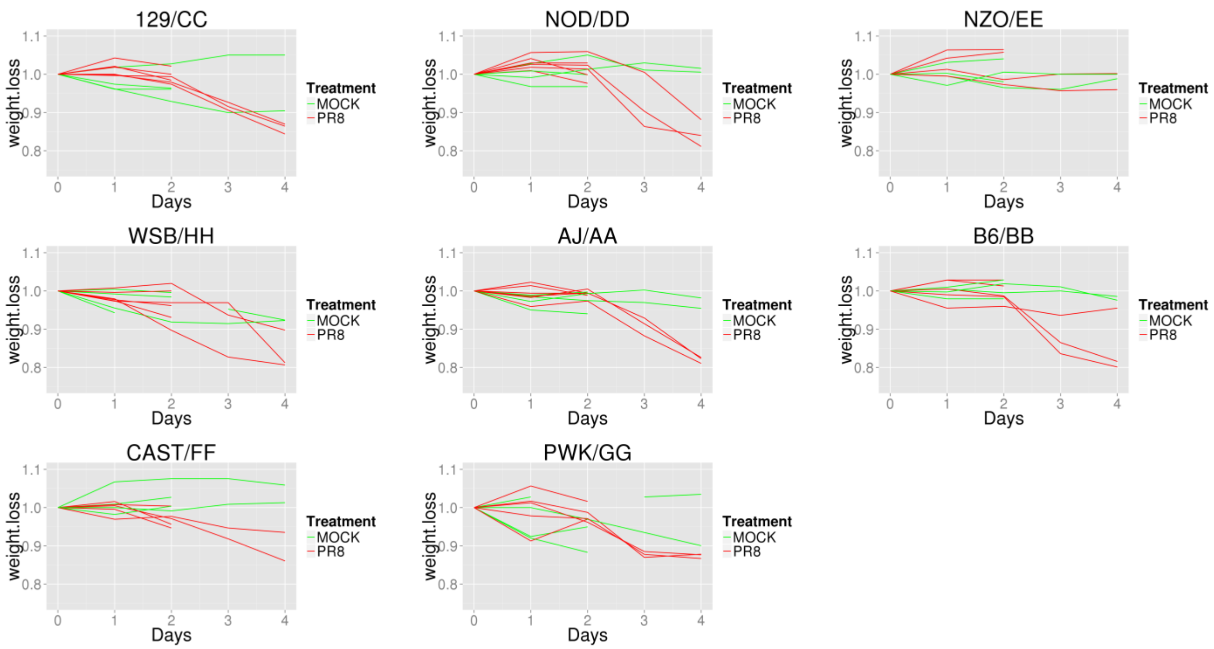

Figure S4 Weight loss of founder mice infected with PR8. Mice were infected with 5 × 10^2 PFU of PR8 and monitored for weight loss for 4 days. Half of the infected animals were sacrificed for expression profiles at day 2 post infection, while the second half were sacrificed at day 4 post infection. There was little weight loss and no discernable strain difference on day 2 post infection. On day 4, NZO had little to no weight loss while all other strains have appreciable weight loss, with most samples showing 10% loss. Half of infected animals were sacrificed for expression profiles at day 2 post-infect while the second half was sacrificed at day 4.

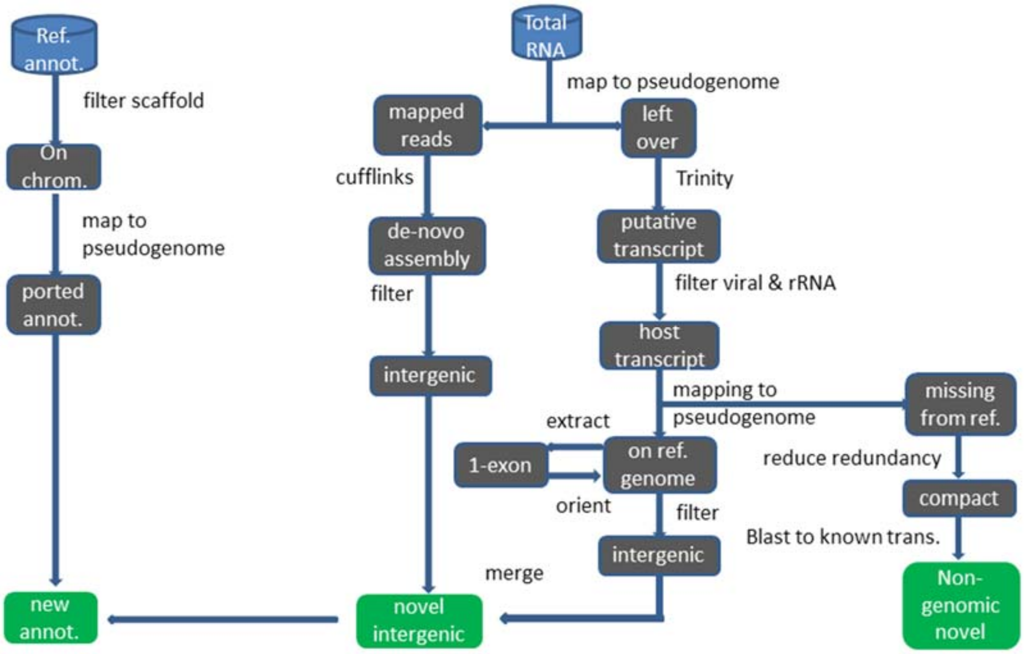

Figure S5   Schematic of the annotation pipeline. The left branch adapts the reference annotation to eight founders' genomes, since the reference annotation is based on C57BL/6J mouse's genome. Before adapting the reference annotation, genes not on chromosomes but on scaffolds are filtered out because by definition they cannot be placed on chromosomes. The right branch is for novel transcript discovery. There are two complementary approaches to discovering transcripts: genome-based and non-genome-based. Cufflinks, a *de novo* assembler, was used for the genome-based approach, and Trinity, another *de novo* assembler, does not require a genome for novel transcripts discovery. After several post-processing steps (see Methods) are condcuted with Trinity, output is merged with Cufflinks' output to obtain novel transcripts. An extra step of filtering viral and ribosomal sequences was added to remove any residual viral and ribosomal sequences due to the weaker mappability of short reads compared to the assembled contigs. Using this approach some *de novo* transcripts could not be placed on existing mouse genomes. After reducing redundancy between *de novo* transcripts, we filter out transcripts similar to sequences on scaffolds and organisms other than human and mouse. There is also an additional stage for orienting single-exon transcripts. The final product of this pipeline was a new annotation with novel transcripts.

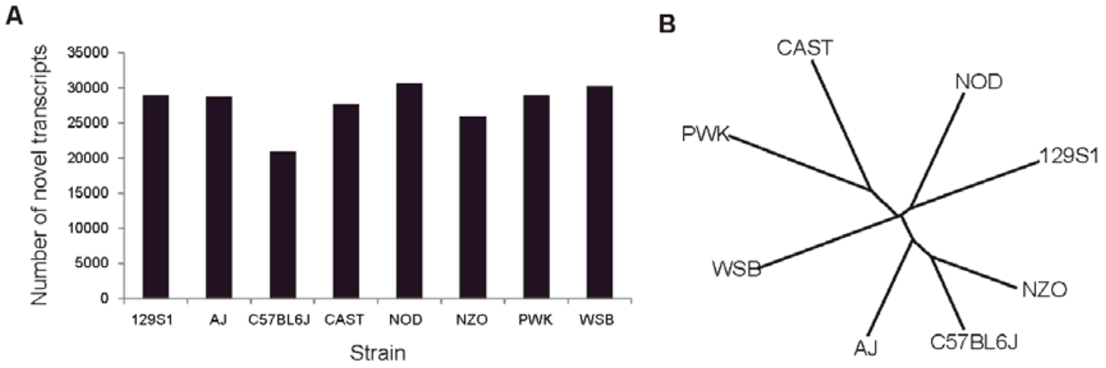

Figure S6 Summary of novel transcripts in eight founder strains and their relationships. (A) the number of novel intergenic novel transcripts in the eight founders. C57BL/6J had the fewest new transcripts because the reference annotation is based on data on the same strain and therefore is most complete for this strain. Other strains had more new intergenic transcripts but wild-derived strains did not appear to possess more than laboratory strains (except C57BL/6J). (B) clustering of founder strains by chromosome 2 novel-transcript density. The clustering results are to the phylogeny of the eight mouse strains. PWK and CAST are separate from all other six strains while WSB is the wild-derived strain closest to the classical laboratory strains.

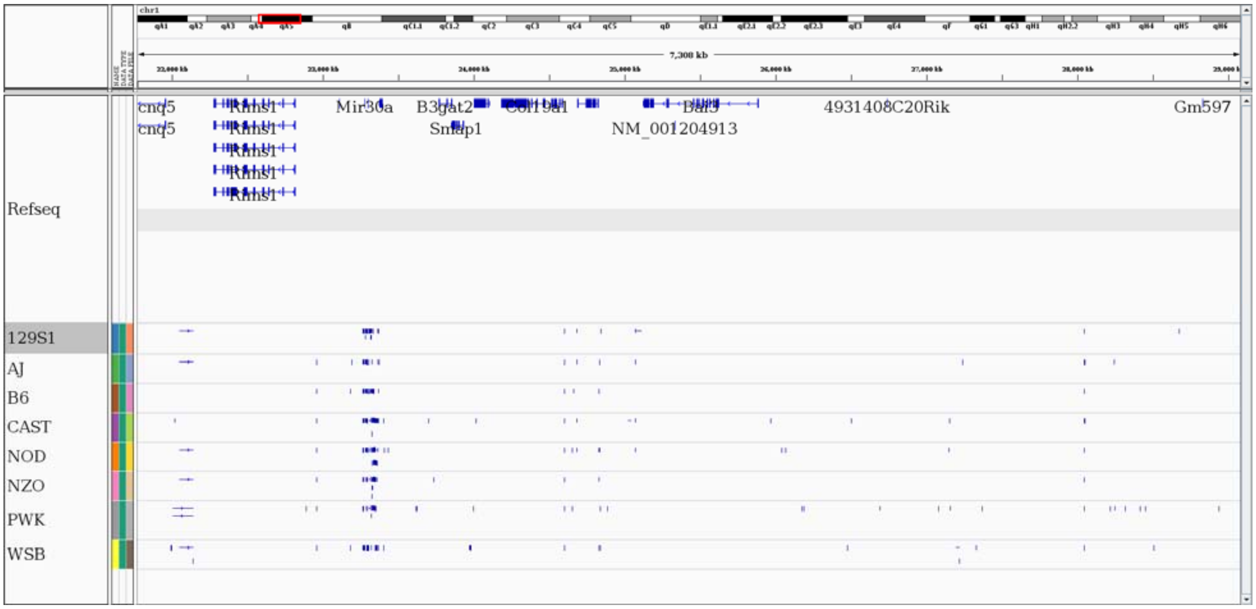

Figure S7 An IGV view of annotated and novel transcripts within HrI3 (chr1:21,767,867—29,085,401) which was found to be associated with pulmonary edema in mice infected with PR8 (Ferris et al. 2013). The top half are the annotated genes from the reference annotation while the rest shows novel transcripts in eight founders. The cluster of novel transcripts near Mir30a could herald previously unknown genes or considerable extension of nearby genes. It is also easy to similarity between the eight founders as well as divergence.

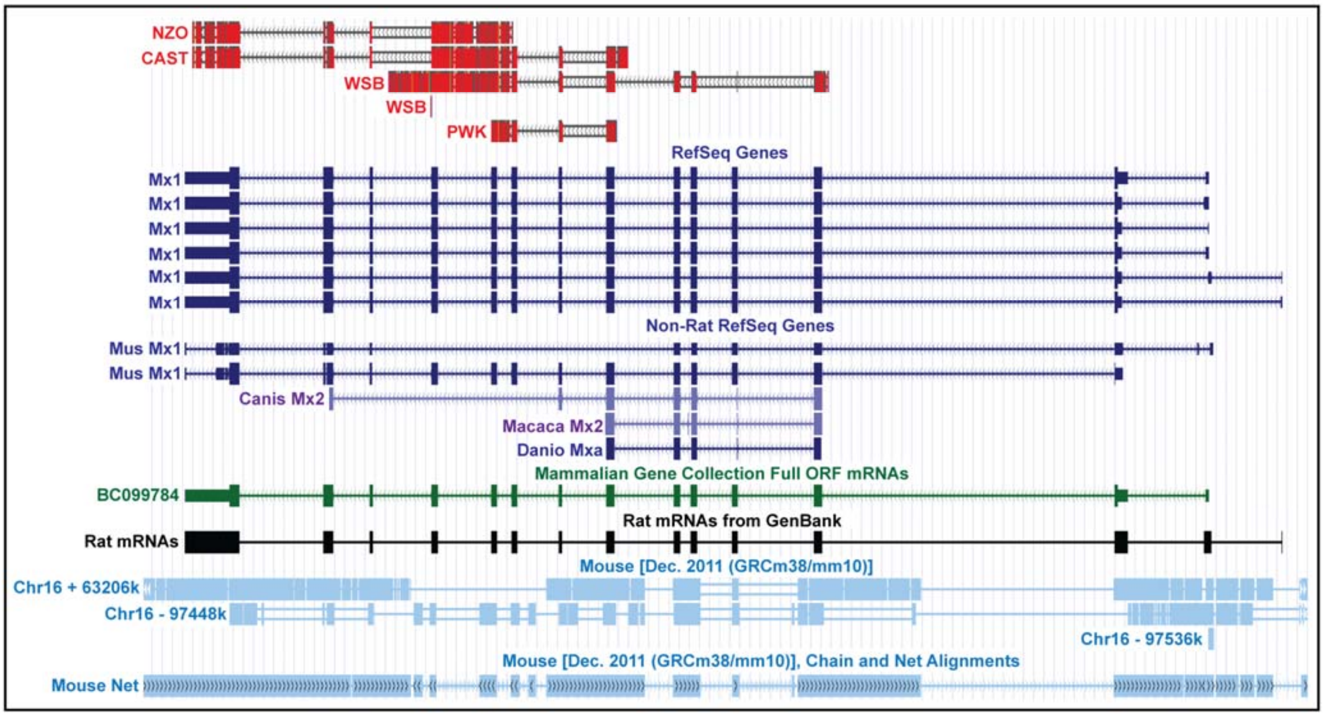

Figure S8 Alignment of de novo transcript with affinity to four extra *Mx1* exons. Ferris et al reported that four founder strains have extra exons in *Mx1* gene compared to the reference annotation. We therefore mapped our novel transcripts against these extra exons and found four novel transcripts containing all of parts of these exons in the four previously reported strains. Mapping the four novel transcripts to rat genome found was successful and shown here. The four transcripts were found in the four strains that Ferris et al originally discovered the extra exons. The other four strains didn't have these missing exons and therefore had no novel transcript that could be mapped here.

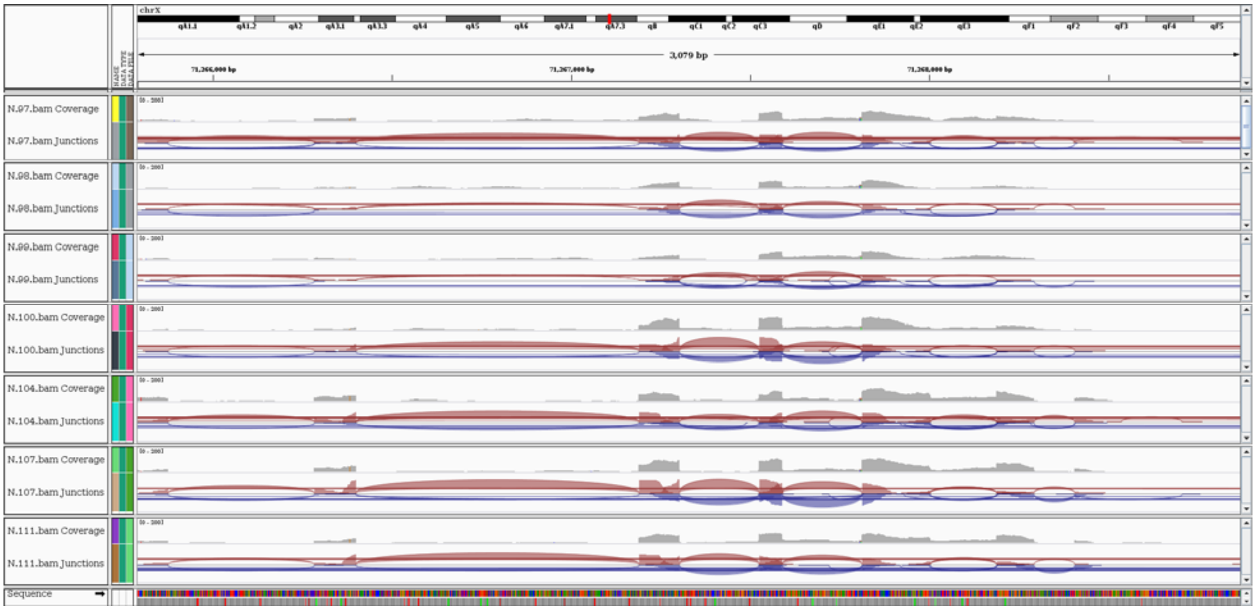

Figure S9 Differential isoform expression of Irak1 gene in MA15-infected CAST mice at day 2 post- infection by MA15. The rightmost splicing junction was differentially expressed: the infected samples (top three) had much lower level of expression than mock samples (bottom four). The gene overall was not differentially expressed as can be seen in the coverage graphs.

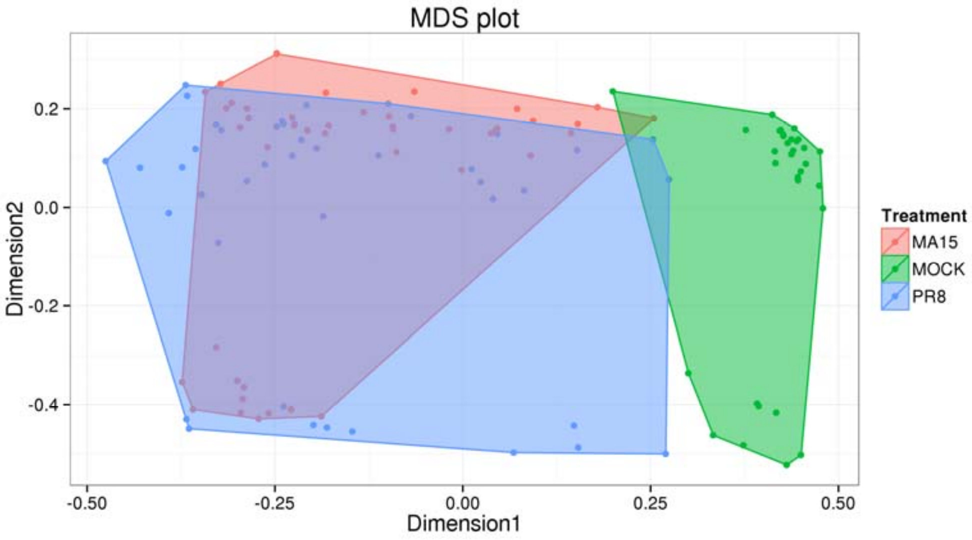

Figure S10 MDS plot of all 119 lung samples that were analyzed for differential expression. Mock samples are colored green, MA15 infection samples are red, and PR8 infection samples are blue. The same colors are also used to define the space spanned by the respective samples.

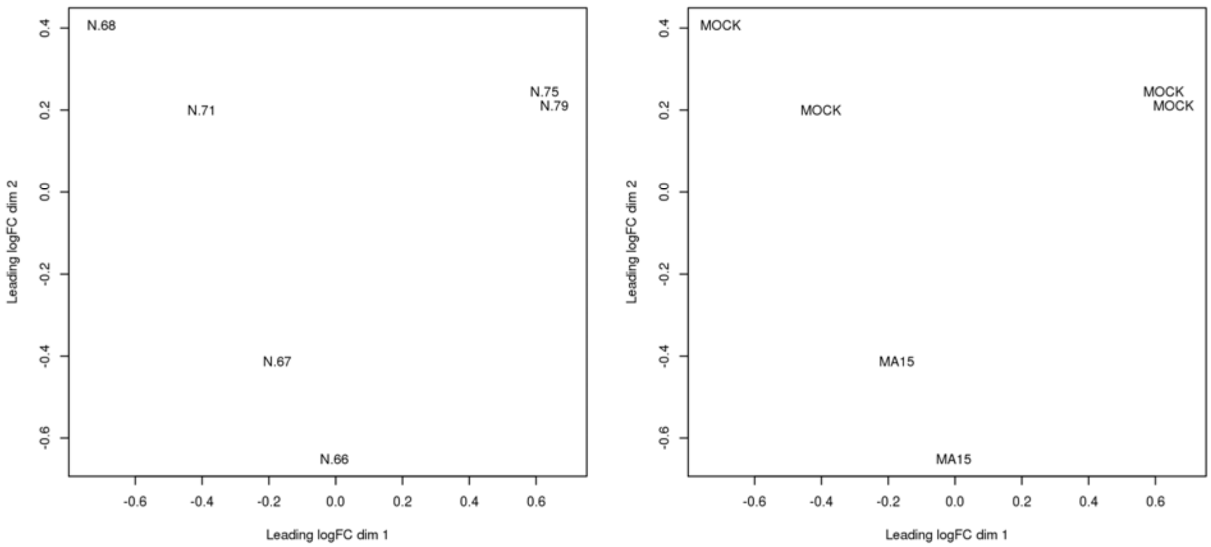

Figure S11 The MDS plot of six samples for AJ strain infected with MA15 at day 2 post infection. The two subplots have different text but are based on the same data. MDS plot is used to gauge possible batch-effect and separation between mocks and infected samples, which are well separated.

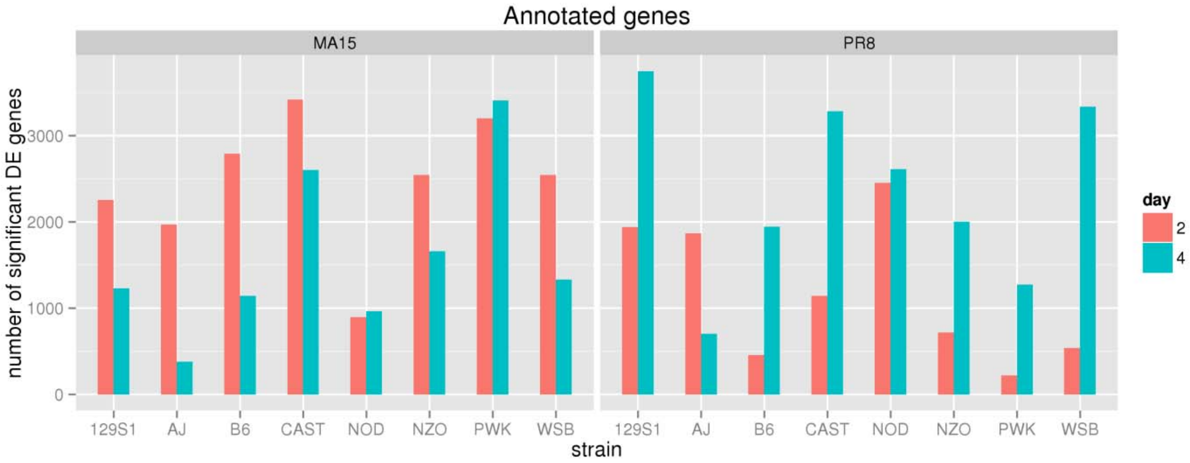

Figure S12 Differentially expressed genes among the eight founder mouse strains following either influenza or SARS-CoV infection. The counts for two viruses were separated into two panels, the left panel for MA15 and the right panel for PR8. Red bars depict DE genes for day 2 post-infection and blue bars depict DE genes for day 4 post-infection.

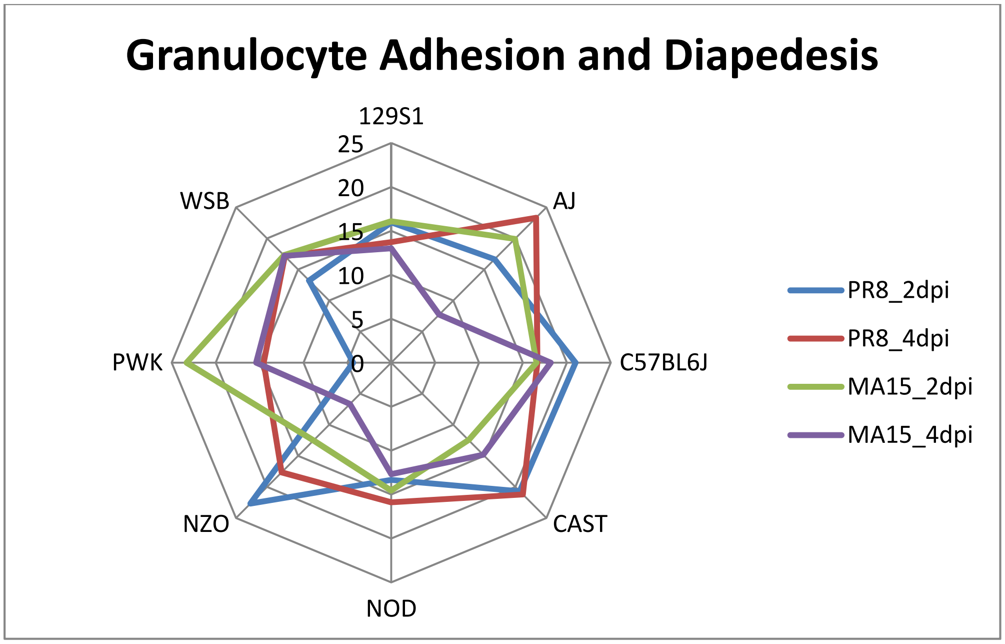

Figure S13 Radial plot showing functional enrichment of granulocyte adhesion and diapedesis pathway genes. This pathway is related to a vascular cuffing phenotype. The strain difference was quite pronounced for both viruses and time points except PR8 on day 4 post-infection. At day 2 post influenza infection the biggest contrast is between PWK and NZO strain, between no enrichment and extreme enrichment. For SARs infection, the difference isn't so big but they were clearly visible. At day 2 post infection, NZO and PWK strains showed mild and strong enrichment, respectively; and at day 4 NZO and A/J strains had less enrichment than other strains.

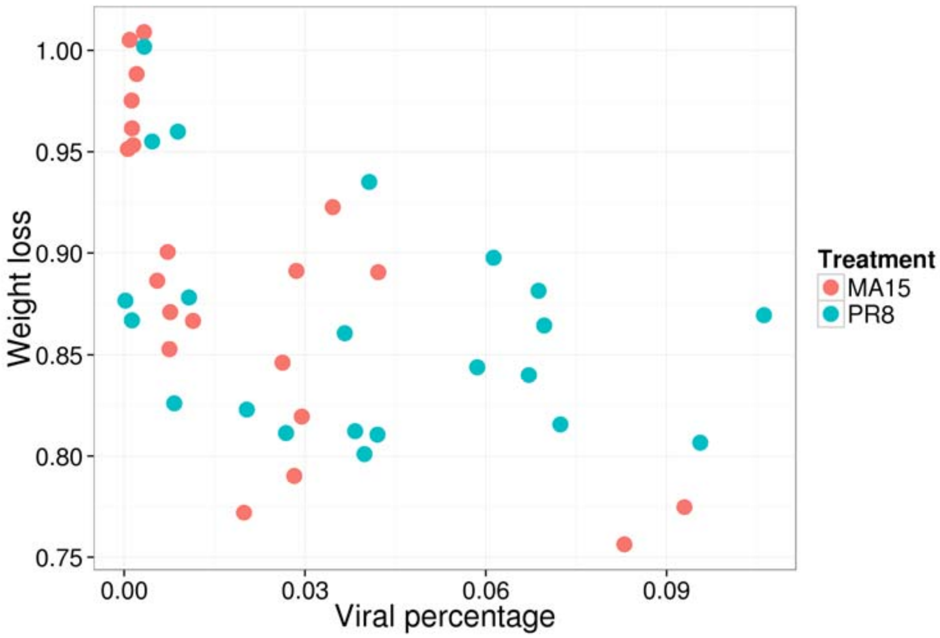

Figure S14 Percent weight loss correlates with viral read percentages. The viral read percentages were plotted against day 4 weight loss. All infected samples were included, with the two viral infections represented by different colored points.

Table S1 The number of splice junctions that were discovered in one strain but not in another strain. One strain (in the row heading) is compared to another strain (in the column heading). For example, there are 3515 junctions in 129S1 that are absent from AJ.

	**129S1**	**AJ**	**B6**	**CAST**	**NOD**	**NZO**	**PWK**	**WSB**
**129S1**	0	3515	10824	14581	3279	7901	8250	7576
**AJ**	6507	0	13508	17474	4980	9888	9294	9925
**B6**	1421	1113	0	7877	974	2526	4363	2976
**CAST**	3156	3057	5855	0	2957	4556	4746	4101
**NOD**	5309	4018	12407	16412	0	8954	9255	9108
**NZO**	3187	2182	7215	11267	2210	0	7140	5255
**PWK**	11641	9693	17157	19562	10616	15245	0	14535
**WSB**	3154	2511	7957	11104	2656	5547	6722	0

Table S2 The number of splice junctions that are observed in infected samples but not in mock samples. Thresholds were set at 10 reads for infected samples and 5 reads for mock samples.

	MA15		PR8	
	**Day 2**	**Day4**	**Day 2**	**Day 4**
**129S1**	2114	1868	2854	4504
**AJ**	4615	1818	2674	788
**B6**	2344	757	371	7877
**CAST**	1487	2666	2078	3934
**NOD**	2294	3173	1825	2496
**NZO**	1844	2827	4353	3562
**PWK**	4675	4093	429	2455
**WSB**	2842	2377	6359	4616

Table S3 The number of differentially expressed splicing junctions. Here a DE junction must be outside DE genes to disambiguate isoform DE from gene DE. There is one order of magnitude of difference between different strains or time points. We took a decidedly conservative approach. There likely are more differentially expressed splicing junctions than we found.

	**MA15**		**PR8**	
**Strain**	**Day 2**	**Day 4**	**Day 2**	**Day 4**
**129S1**	453	326	219	720
**AJ**	25	237	306	174
**B6**	817	183	42	628
**CAST**	855	581	192	783
**NOD**	206	282	521	438
**NZO**	612	184	101	268
**PWK**	692	976	64	235
**WSB**	964	276	75	822

Table S4 The replicate size for each combination of mouse strain, virus, and days-post-infection. Note that mocks are pooled for all DE analysis.

Strain	Virus	Day	Sample size
**129S1**	MA15	2	3
**129S1**	MA15	4	3
**129S1**	MOCK	2	2
**129S1**	MOCK	4	2
**129S1**	PR8	2	3
**129S1**	PR8	4	3
**AJ**	MA15	2	3
**AJ**	MA15	4	2
**AJ**	MOCK	2	2
**AJ**	MOCK	4	2
**AJ**	PR8	2	3
**AJ**	PR8	4	3
**B6**	MA15	2	3
**B6**	MA15	4	3
**B6**	MOCK	2	2
**B6**	MOCK	4	2
**B6**	PR8	2	2
**B6**	PR8	4	3
**CAST**	MA15	2	3
**CAST**	MA15	4	2
**CAST**	MOCK	2	2
**CAST**	MOCK	4	2
**CAST**	PR8	2	3
**CAST**	PR8	4	2
**NOD**	MA15	2	3
**NOD**	MA15	4	3
**NOD**	MOCK	2	2
**NOD**	MOCK	4	2
**NOD**	PR8	2	3
**NOD**	PR8	4	3
**NZO**	MA15	2	2
**NZO**	MA15	4	2
**NZO**	MOCK	2	2
**NZO**	MOCK	4	2
**NZO**	PR8	2	2
**NZO**	PR8	4	2
**PWK**	MA15	2	3
**PWK**	MA15	4	3
**PWK**	MOCK	2	2

**PWK**	MOCK	4	2
**PWK**	PR8	2	2
**PWK**	PR8	4	3
**WSB**	MA15	2	3
**WSB**	MA15	4	3
**WSB**	MOCK	2	2
**WSB**	MOCK	4	2
**WSB**	PR8	2	3
**WSB**	PR8	4	3

Table S5 Viral read counts in the mock-infected founders. Each strain's total comes from four mice. The counts are low, several orders of magnitude lower than infected samples. The fact we still get non-zero reads may be an artifact of mapping and select strains may have partial sequence similarity to viruses.

Strain	MA15	PR8
**129S1**	5084	11394
**AJ**	9549	2659
**B6**	521	1656
**CAST**	10490	3060
**NOD**	608	857
**NZO**	629	636
**PWK**	957	634
**WSB**	144	7491
