# Supplementary material for: Genomic Profiling of Collaborative Cross Founder Mice Infected with Respiratory Viruses Reveals Novel Transcripts and Infection-Related Strain-Specific Gene and Isoform Expression
Source: G3 (Bethesda). 2014 Jun 5;4(8):1429–44. doi: 10.1534/g3.114.011759 (PMC4132174; doi:10.1534/g3.114.011759)
Supplement: Supporting Information [file supp_g3.114.011759_FileS1.pdf]

File S1

Transcript discovery pipeline

The transcript discovery pipeline accomplishes two tasks, adapting existing gene models to the different founder genomes and uncovering new transcripts not overlapping with any known transcripts. To use the most accurate genome for each founders we downloaded the pseudogenomes that had been generated by University of North Carolina based on DNA-seq data produced by the Sanger Institute. This incorporates single-nucleotide variations (SNV), indels, and other structural variations, which can number in the millions, and represents our most current description of the founder genomes. The reference annotation describes over thirty thousand genes, of which about twenty four thousand genes are coding genes. We mapped short reads to their respective pseudogenomes and used the Cufflinks program to predict new transcripts. To avoid confounding intronic reads, we retained only intergenic transcripts. We also took unmapped reads and used Trinity to discover *de novo* transcripts. After filtering *de novo* transcripts that bore resemblance to viral or host genomes, we combined the Trinity output that mapped to mouse genomes with the Cufflinks output, while the stand alone *de novo* transcripts were further filtered to identifythose that were similar to known mouse, rat, or human sequences. The outline of our novel transcript discovery pipeline built is shown in Figure S5.
