# Supplementary material for: Genomic Profiling of Collaborative Cross Founder Mice Infected with Respiratory Viruses Reveals Novel Transcripts and Infection-Related Strain-Specific Gene and Isoform Expression
Source: G3 (Bethesda). 2014 Jun 5;4(8):1429–44. doi: 10.1534/g3.114.011759 (PMC4132174; doi:10.1534/g3.114.011759)
Supplement: Supporting Information [file supp_g3.114.011759_FigureS1.pdf]

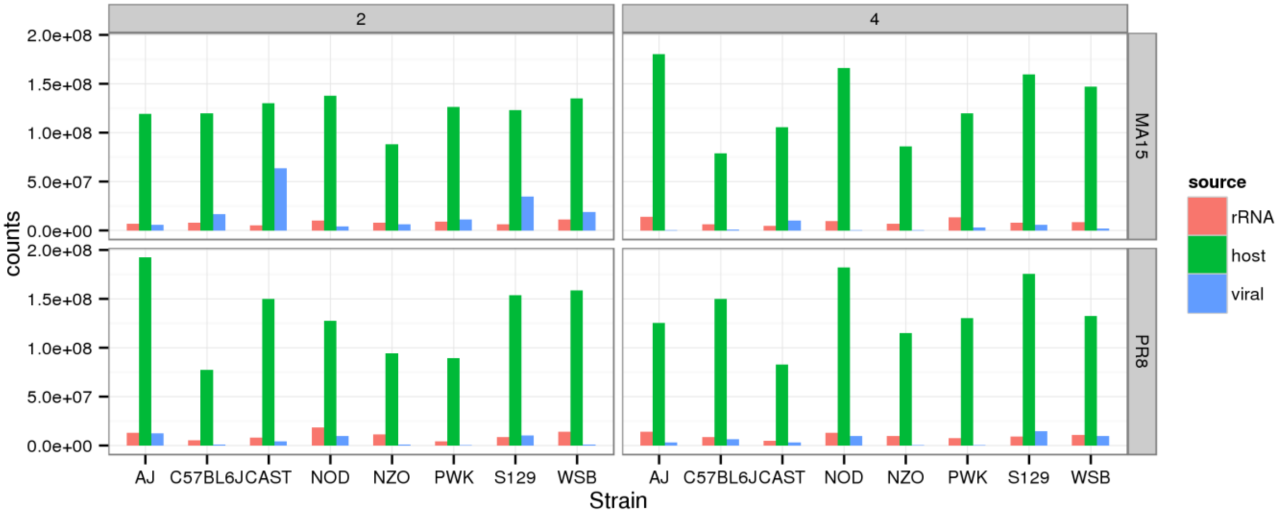

Figure S1 The number of short RNA reads that map to host, ribosomes, and viruses. The read counts were summed by the unique combination of mouse strain, virus, and day post infection. We generally ensured each sample has at least 30 million host reads. The varying sequencing depth for ribosomal and host RNA among strains resulted from random sequencing variation. On the other hand, the viral read counts exhibited strain specificity. For example, MA15-infected CAST mice had 60M viral reads on day 2, while MA15-infected NZO mice had about 6M viral reads, which pointed to strain differences in viral replication.
