# Supplementary material for: Genomic Profiling of Collaborative Cross Founder Mice Infected with Respiratory Viruses Reveals Novel Transcripts and Infection-Related Strain-Specific Gene and Isoform Expression
Source: G3 (Bethesda). 2014 Jun 5;4(8):1429–44. doi: 10.1534/g3.114.011759 (PMC4132174; doi:10.1534/g3.114.011759)
Supplement: Supporting Information [file supp_g3.114.011759_FigureS2.pdf]

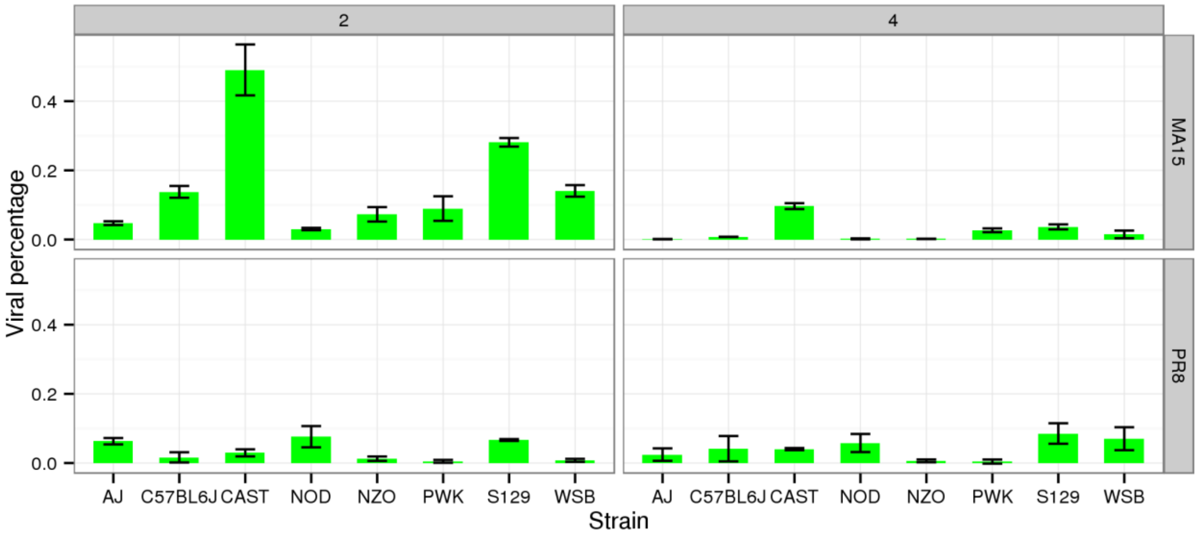

Figure S2 The percentage of RNA reads that belong to MA15 or PR8 genomes. The percentages were calculated by summing all viral reads from animals infected with one virus type for each founder strain at different time points (2 and 4 days post infection) and divided by the total read counts. Each strain had at least two replicates and most have three replicates. There are clear strain-specific differences in the viral read percentages, ranging from almost zero percent of RNA reads in MA15-infected AJ mice to close to half of reads in MA15-infected CAST mice on day 2 post infection.
