# Supplementary material for: Genomic Profiling of Collaborative Cross Founder Mice Infected with Respiratory Viruses Reveals Novel Transcripts and Infection-Related Strain-Specific Gene and Isoform Expression
Source: G3 (Bethesda). 2014 Jun 5;4(8):1429–44. doi: 10.1534/g3.114.011759 (PMC4132174; doi:10.1534/g3.114.011759)
Supplement: Supporting Information [file supp_g3.114.011759_FigureS3.pdf]

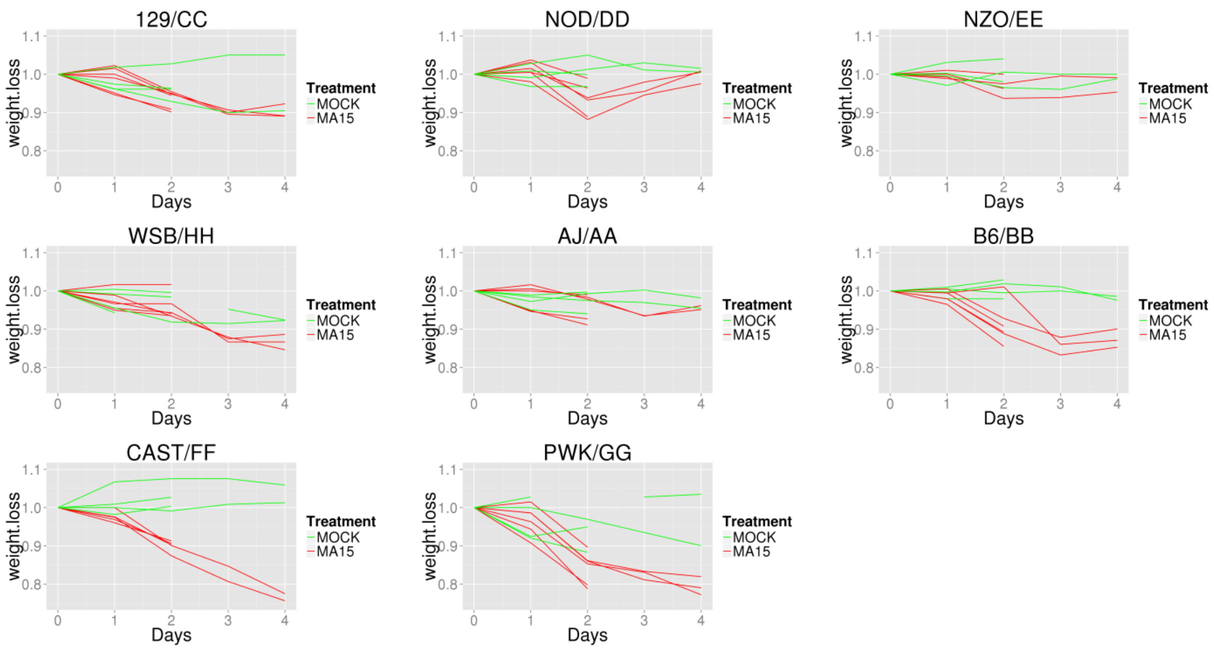

Figure S3 Weight loss of founder mice infected with MA15. Mice were infected with 10^5 PFU of MA15 and monitored for weight loss for 4 days. Half of the infected animals were sacrificed for expression profiles at day 2 post- nfection, while the second half were sacrificed at day 4 post infection. In general, there were observable strain differences in weight loss. For example, NZO lost less than 5% weight, while NOD and AJ mice were able to regain their lost weight by day 4. In contrast, the remaining mouse strains sustained about 10% weight loss, with the CAST and PWK strains showing close to 20% weight loss.
