# Supplementary material for: Genomic Profiling of Collaborative Cross Founder Mice Infected with Respiratory Viruses Reveals Novel Transcripts and Infection-Related Strain-Specific Gene and Isoform Expression
Source: G3 (Bethesda). 2014 Jun 5;4(8):1429–44. doi: 10.1534/g3.114.011759 (PMC4132174; doi:10.1534/g3.114.011759)
Supplement: Supporting Information [file supp_g3.114.011759_FigureS4.pdf]

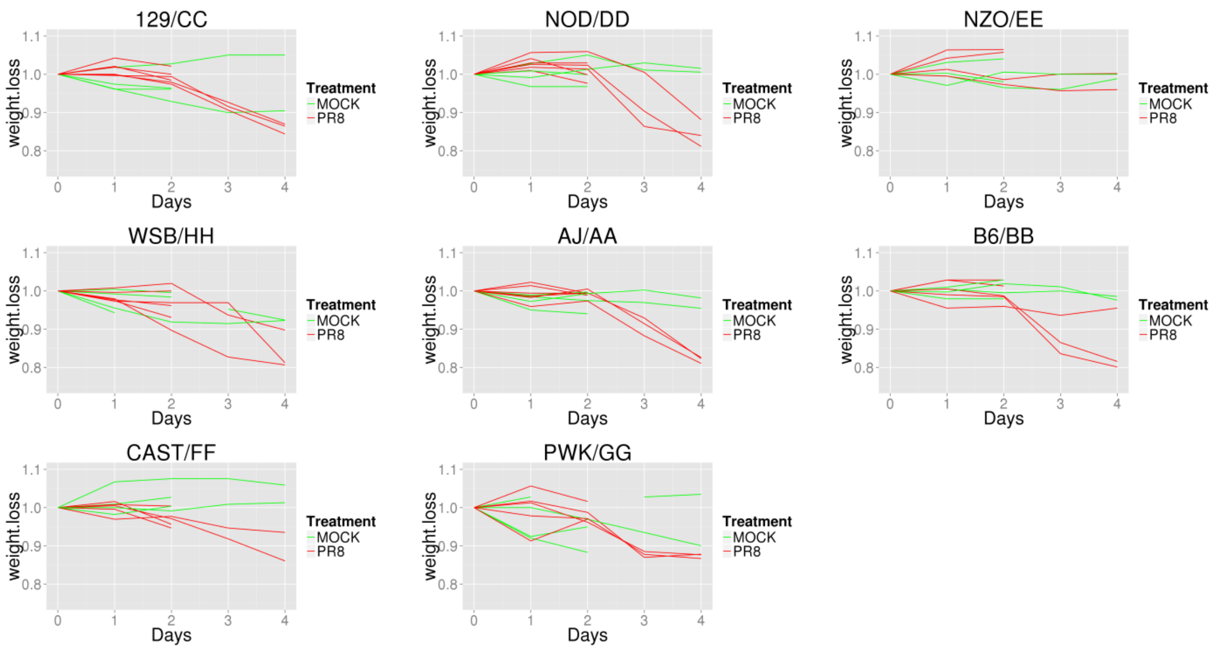

Figure S4 Weight loss of founder mice infected with PR8. Mice were infected with 5 × 10^2 PFU of PR8 and monitored for weight loss for 4 days. Half of the infected animals were sacrificed for expression profiles at day 2 post infection, while the second half were sacrificed at day 4 post infection. There was little weight loss and no discernable strain difference on day 2 post infection. On day 4, NZO had little to no weight loss while all other strains have appreciable weight loss, with most samples showing 10% loss. Half of infected animals were sacrificed for expression profiles at day 2 post-infect while the second half was sacrificed at day 4.
