# Supplementary material for: Genomic Profiling of Collaborative Cross Founder Mice Infected with Respiratory Viruses Reveals Novel Transcripts and Infection-Related Strain-Specific Gene and Isoform Expression
Source: G3 (Bethesda). 2014 Jun 5;4(8):1429–44. doi: 10.1534/g3.114.011759 (PMC4132174; doi:10.1534/g3.114.011759)
Supplement: Supporting Information [file supp_g3.114.011759_FigureS5.pdf]

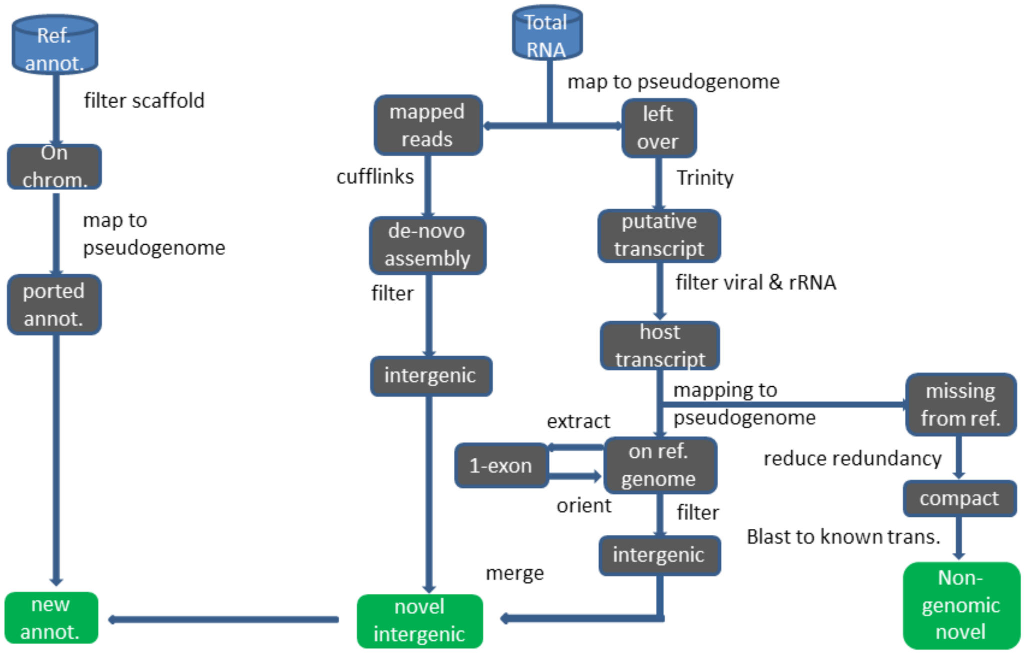

Figure S5 Schematic of the annotation pipeline. The left branch adapts the reference annotation to eight founders' genomes, since the reference annotation is based on C57BL/6J mouse's genome. Before adapting the reference annotation, genes not on chromosomes but on scaffolds are filtered out because by definition they cannot be placed on chromosomes. The right branch is for novel transcript discovery. There are two complementary approaches to discovering transcripts: genome-based and non-genome-based. Cufflinks, a *de novo* assembler, was used for the genome-based approach, and Trinity, another *de novo* assembler, does not require a genome for novel transcripts discovery. After several post-processing steps (see Methods) are condcuted with Trinity, output is merged with Cufflinks' output to obtain novel transcripts. An extra step of filtering viral and ribosomal sequences was added to remove any residual viral and ribosomal sequences due to the weaker mappability of short reads compared to the assembled contigs. Using this approach some *de novo* transcripts could not be placed on existing mouse genomes. After reducing redundancy between *de novo* transcripts, we filter out transcripts similar to sequences on scaffolds and organisms other than human and mouse. There is also an additional stage for orienting single-exon transcripts. The final product of this pipeline was a new annotation with novel transcripts.
