# Supplementary material for: Genomic Profiling of Collaborative Cross Founder Mice Infected with Respiratory Viruses Reveals Novel Transcripts and Infection-Related Strain-Specific Gene and Isoform Expression
Source: G3 (Bethesda). 2014 Jun 5;4(8):1429–44. doi: 10.1534/g3.114.011759 (PMC4132174; doi:10.1534/g3.114.011759)
Supplement: Supporting Information [file supp_g3.114.011759_FigureS6.pdf]

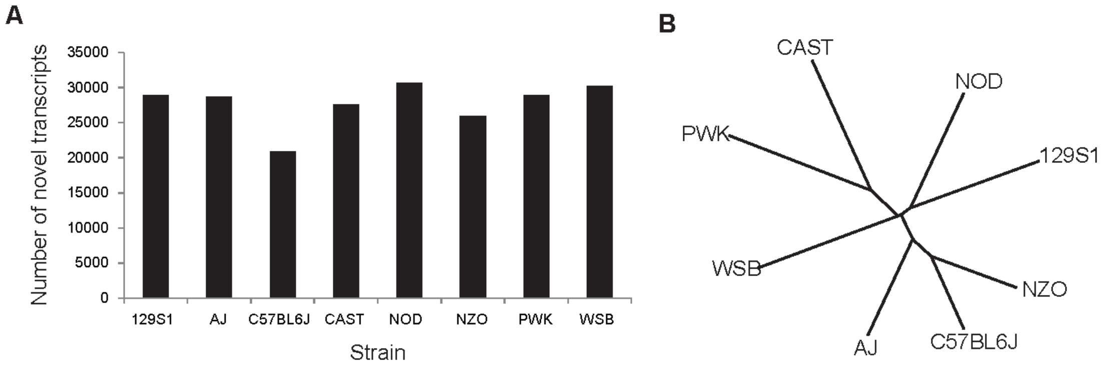

Figure S6 Summary of novel transcripts in eight founder strains and their relationships. (A) the number of novel intergenic novel transcripts in the eight founders. C57BL/6J had the fewest new transcripts because the reference annotation is based on data on the same strain and therefore is most complete for this strain. Other strains had more new intergenic transcripts but wild-derived strains did not appear to possess more than laboratory strains (except C57BL/6J). (B) clustering of founder strains by chromosome 2 novel-transcript density. The clustering results are to the phylogeny of the eight mouse strains. PWK and CAST are separate from all other six strains while WSB is the wild-derived strain closest to the classical laboratory strains.
