# Supplementary material for: Genomic Profiling of Collaborative Cross Founder Mice Infected with Respiratory Viruses Reveals Novel Transcripts and Infection-Related Strain-Specific Gene and Isoform Expression
Source: G3 (Bethesda). 2014 Jun 5;4(8):1429–44. doi: 10.1534/g3.114.011759 (PMC4132174; doi:10.1534/g3.114.011759)
Supplement: Supporting Information [file supp_g3.114.011759_FigureS7.pdf]

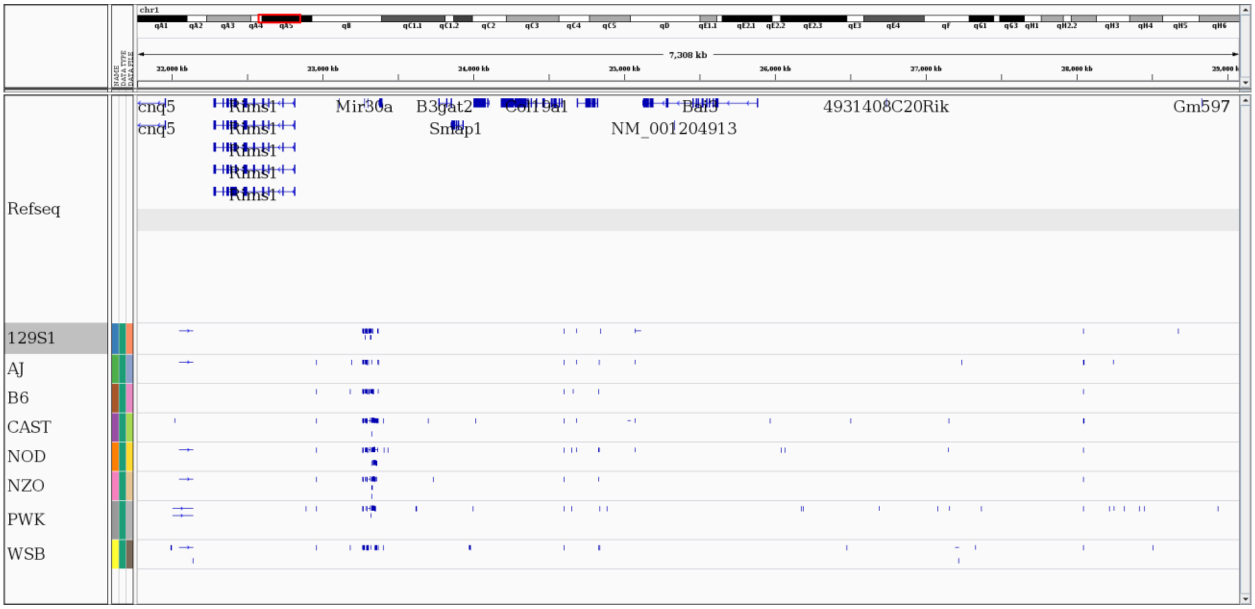

Figure S7 An IGV view of annotated and novel transcripts within Hrl3 (chr1:21,767,867—29,085,401) which was found to be associated with pulmonary edema in mice infected with PR8 (Ferris et al. 2013). The top half are the annotated genes from the reference annotation while the rest shows novel transcripts in eight founders. The cluster of novel transcripts near Mir30a could herald previously unknown genes or considerable extension of nearby genes. It is also easy to similarity between the eight founders as well as divergence.
