# Supplementary material for: Genomic Profiling of Collaborative Cross Founder Mice Infected with Respiratory Viruses Reveals Novel Transcripts and Infection-Related Strain-Specific Gene and Isoform Expression
Source: G3 (Bethesda). 2014 Jun 5;4(8):1429–44. doi: 10.1534/g3.114.011759 (PMC4132174; doi:10.1534/g3.114.011759)
Supplement: Supporting Information [file supp_g3.114.011759_FigureS8.pdf]

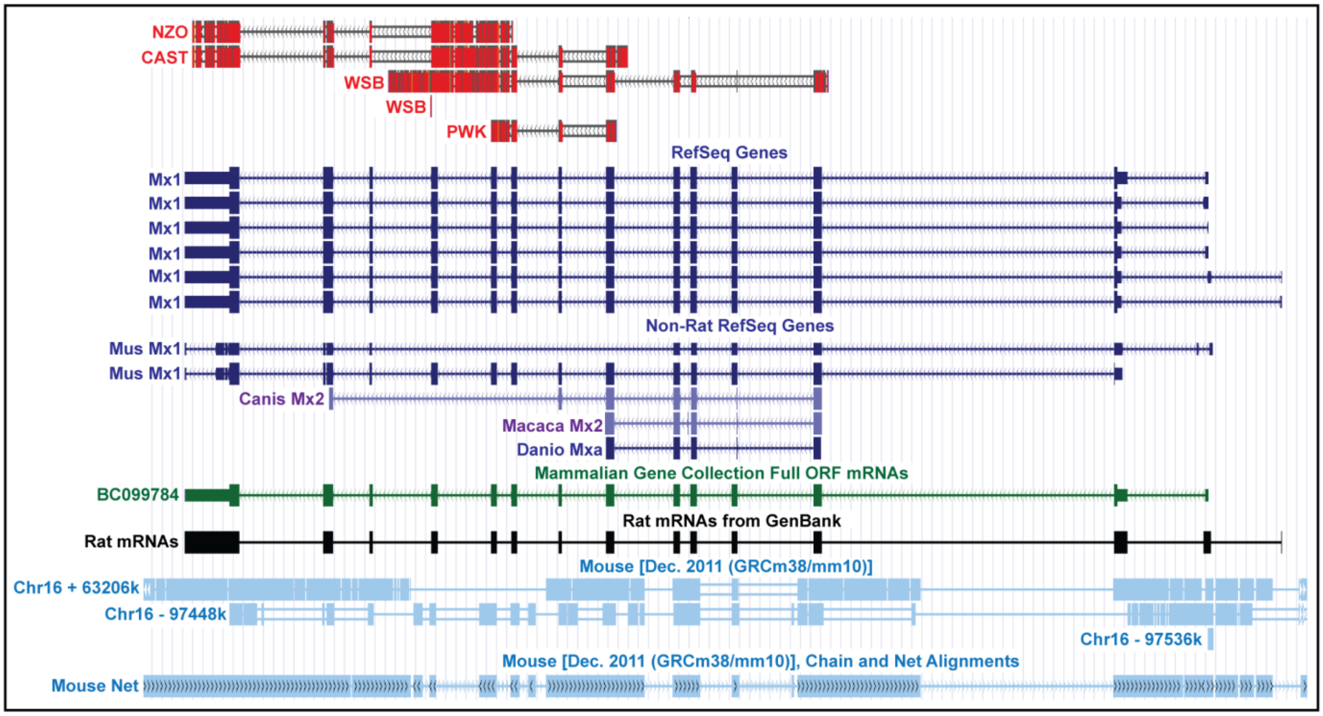

Figure S8 Alignment of de novo transcript with affinity to four extra *Mx1* exons. Ferris et al reported that four founder strains have extra exons in *Mx1* gene compared to the reference annotation. We therefore mapped our novel transcripts against these extra exons and found four novel transcripts containing all of parts of these exons in the four previously reported strains. Mapping the four novel transcripts to rat genome found was successful and shown here. The four transcripts were found in the four strains that Ferris et al originally discovered the extra exons. The other four strains didn't have these missing exons and therefore had no novel transcript that could be mapped here.
