# Supplementary material for: Genomic Profiling of Collaborative Cross Founder Mice Infected with Respiratory Viruses Reveals Novel Transcripts and Infection-Related Strain-Specific Gene and Isoform Expression
Source: G3 (Bethesda). 2014 Jun 5;4(8):1429–44. doi: 10.1534/g3.114.011759 (PMC4132174; doi:10.1534/g3.114.011759)
Supplement: Supporting Information [file supp_g3.114.011759_FigureS9.pdf]

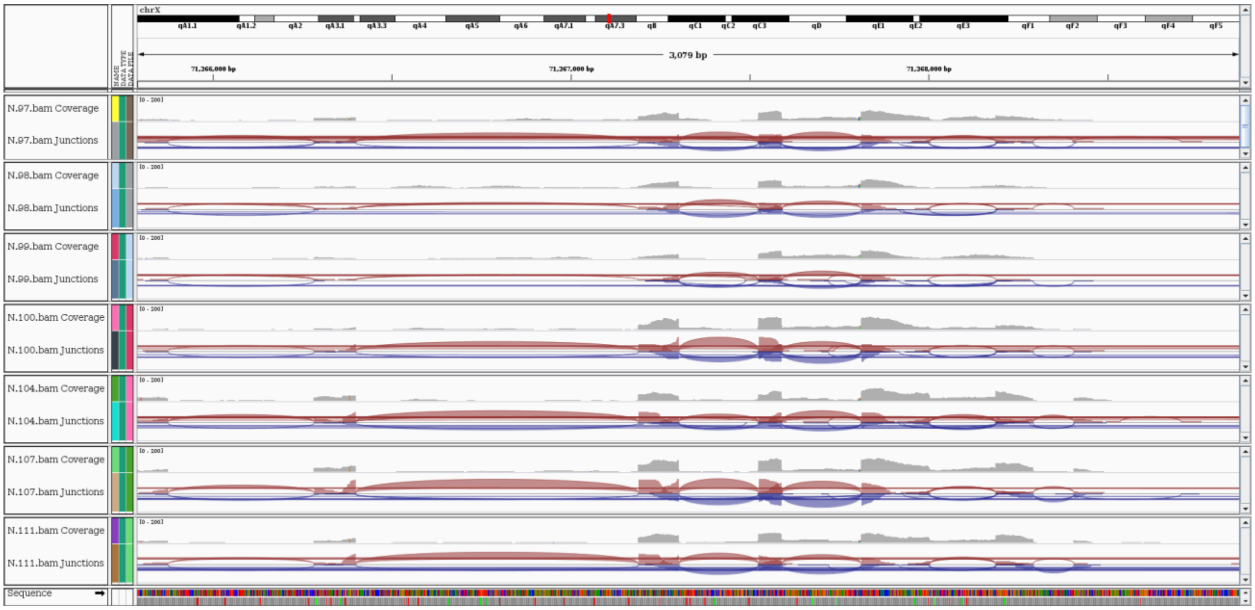

Figure S9 Differential isoform expression of Irak1 gene in MA15-infected CAST mice at day 2 post- infection by MA15. The rightmost splicing junction was differentially expressed: the infected samples (top three) had much lower level of expression than mock samples (bottom four). The gene overall was not differentially expressed as can be seen in the coverage graphs.
