# Supplementary material for: Genomic Profiling of Collaborative Cross Founder Mice Infected with Respiratory Viruses Reveals Novel Transcripts and Infection-Related Strain-Specific Gene and Isoform Expression
Source: G3 (Bethesda). 2014 Jun 5;4(8):1429–44. doi: 10.1534/g3.114.011759 (PMC4132174; doi:10.1534/g3.114.011759)
Supplement: Supporting Information [file supp_g3.114.011759_FigureS10.pdf]

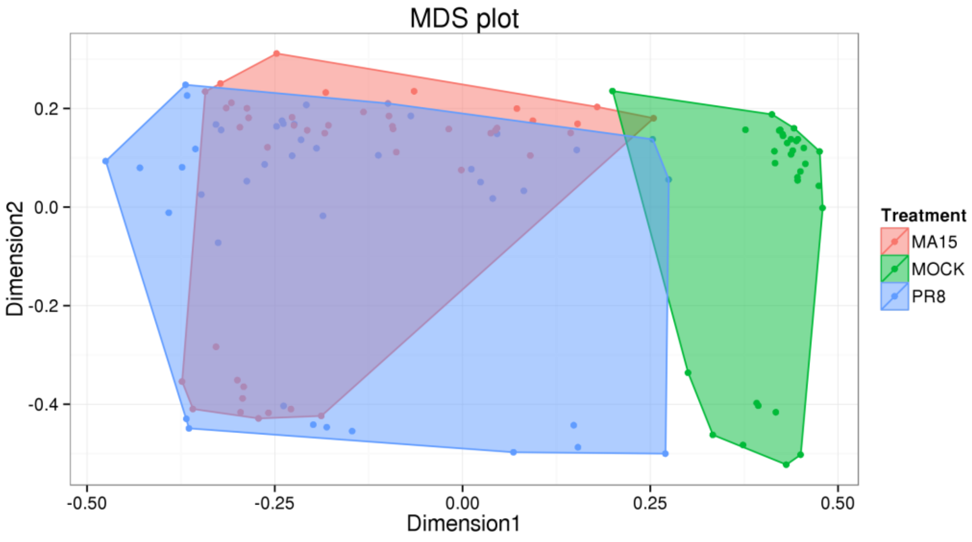

Figure S10 MDS plot of all 119 lung samples that were analyzed for differential expression. Mock samples are colored green, MA15 infection samples are red, and PR8 infection samples are blue. The same colors are also used to define the space spanned by the respective samples.
