# Supplementary material for: Genomic Profiling of Collaborative Cross Founder Mice Infected with Respiratory Viruses Reveals Novel Transcripts and Infection-Related Strain-Specific Gene and Isoform Expression
Source: G3 (Bethesda). 2014 Jun 5;4(8):1429–44. doi: 10.1534/g3.114.011759 (PMC4132174; doi:10.1534/g3.114.011759)
Supplement: Supporting Information [file supp_g3.114.011759_FigureS11.pdf]

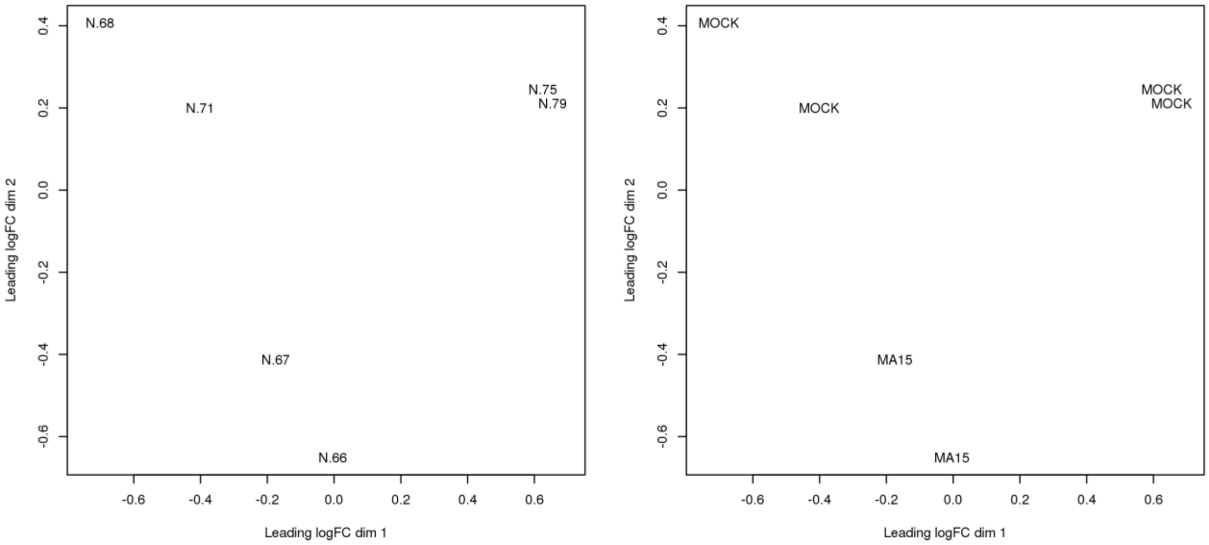

Figure S11 The MDS plot of six samples for AJ strain infected with MA15 at day 2 post infection. The two subplots have different text but are based on the same data. MDS plot is used to gauge possible batch-effect and separation between mocks and infected samples, which are well separated.
