# Supplementary material for: Genomic Profiling of Collaborative Cross Founder Mice Infected with Respiratory Viruses Reveals Novel Transcripts and Infection-Related Strain-Specific Gene and Isoform Expression
Source: G3 (Bethesda). 2014 Jun 5;4(8):1429–44. doi: 10.1534/g3.114.011759 (PMC4132174; doi:10.1534/g3.114.011759)
Supplement: Supporting Information [file supp_g3.114.011759_FigureS12.pdf]

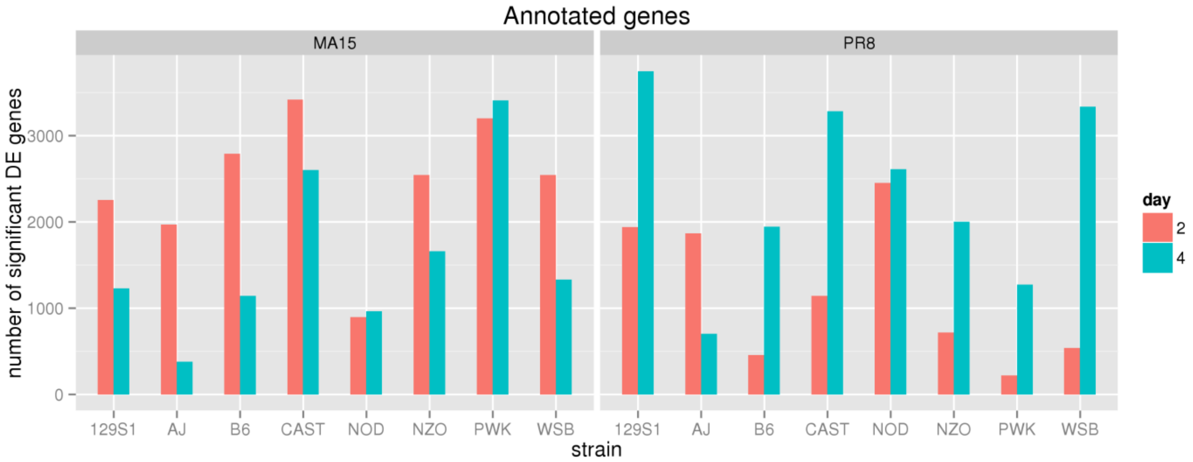

Figure S12 Differentially expressed genes among the eight founder mouse strains following either influenza or SARS-CoV infection. The counts for two viruses were separated into two panels, the left panel for MA15 and the right panel for PR8. Red bars depict DE genes for day 2 post-infection and blue bars depict DE genes for day 4 post-infection.
