# Supplementary material for: Genomic Profiling of Collaborative Cross Founder Mice Infected with Respiratory Viruses Reveals Novel Transcripts and Infection-Related Strain-Specific Gene and Isoform Expression
Source: G3 (Bethesda). 2014 Jun 5;4(8):1429–44. doi: 10.1534/g3.114.011759 (PMC4132174; doi:10.1534/g3.114.011759)
Supplement: Supporting Information [file supp_g3.114.011759_FigureS13.pdf]

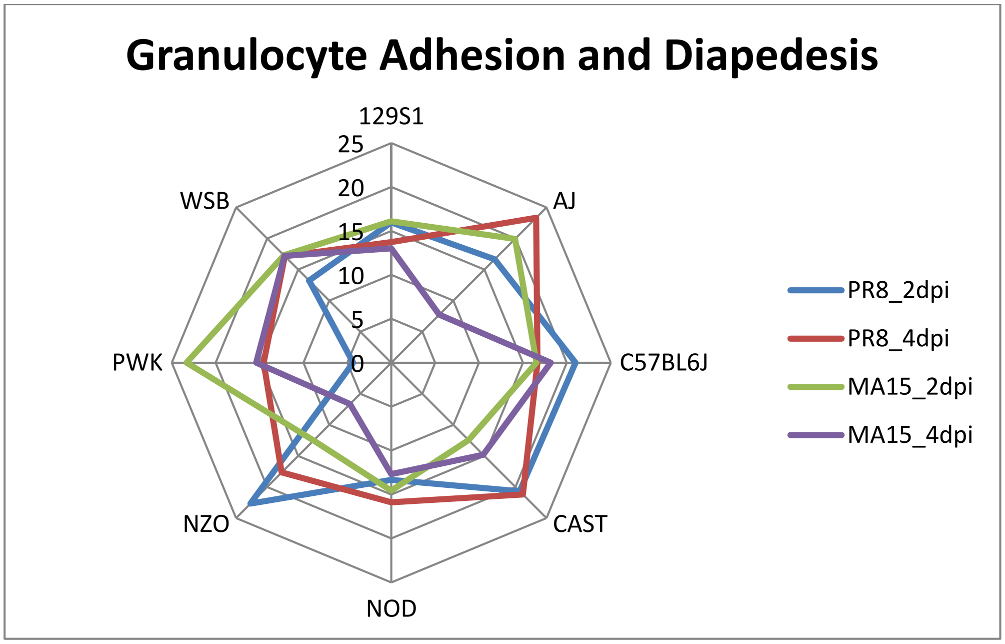

Figure S13 Radial plot showing functional enrichment of granulocyte adhesion and diapedesis pathway genes. This pathway is related to a vascular cuffing phenotype. The strain difference was quite pronounced for both viruses and time points except PR8 on day 4 post-infection. At day 2 post influenza infection the biggest contrast is between PWK and NZO strain, between no enrichment and extreme enrichment. For SARs infection, the difference isn't so big but they were clearly visible. At day 2 post infection, NZO and PWK strains showed mild and strong enrichment, respectively; and at day 4 NZO and A/J strains had less enrichment than other strains.
