# Supplementary material for: Genomic Profiling of Collaborative Cross Founder Mice Infected with Respiratory Viruses Reveals Novel Transcripts and Infection-Related Strain-Specific Gene and Isoform Expression
Source: G3 (Bethesda). 2014 Jun 5;4(8):1429–44. doi: 10.1534/g3.114.011759 (PMC4132174; doi:10.1534/g3.114.011759)
Supplement: Supporting Information [file supp_g3.114.011759_TableS1.pdf]

Table S1 The number of splice junctions that were discovered in one strain but not in another strain. One strain (in the row heading) is compared to another strain (in the column heading). For example, there are 3515 junctions in 129S1 that are absent from AJ.

	**129S1**	**AJ**	**B6**	**CAST**	**NOD**	**NZO**	**PWK**	**WSB**
**129S1**	0	3515	10824	14581	3279	7901	8250	7576
**AJ**	6507	0	13508	17474	4980	9888	9294	9925
**B6**	1421	1113	0	7877	974	2526	4363	2976
**CAST**	3156	3057	5855	0	2957	4556	4746	4101
**NOD**	5309	4018	12407	16412	0	8954	9255	9108
**NZO**	3187	2182	7215	11267	2210	0	7140	5255
**PWK**	11641	9693	17157	19562	10616	15245	0	14535
**WSB**	3154	2511	7957	11104	2656	5547	6722	0
