# Supplementary material for: Genomic Profiling of Collaborative Cross Founder Mice Infected with Respiratory Viruses Reveals Novel Transcripts and Infection-Related Strain-Specific Gene and Isoform Expression
Source: G3 (Bethesda). 2014 Jun 5;4(8):1429–44. doi: 10.1534/g3.114.011759 (PMC4132174; doi:10.1534/g3.114.011759)
Supplement: Supporting Information [file supp_g3.114.011759_TableS2.pdf]

Table S2 The number of splice junctions that are observed in infected samples but not in mock samples. Thresholds were set at 10 reads for infected samples and 5 reads for mock samples.

	**MA15**		**PR8**	
	**Day 2**	**Day4**	**Day 2**	**Day 4**
**129S1**	2114	1868	2854	4504
**AJ**	4615	1818	2674	788
**B6**	2344	757	371	7877
**CAST**	1487	2666	2078	3934
**NOD**	2294	3173	1825	2496
**NZO**	1844	2827	4353	3562
**PWK**	4675	4093	429	2455
**WSB**	2842	2377	6359	4616
