# Supplementary material for: Genomic Profiling of Collaborative Cross Founder Mice Infected with Respiratory Viruses Reveals Novel Transcripts and Infection-Related Strain-Specific Gene and Isoform Expression
Source: G3 (Bethesda). 2014 Jun 5;4(8):1429–44. doi: 10.1534/g3.114.011759 (PMC4132174; doi:10.1534/g3.114.011759)
Supplement: Supporting Information [file supp_g3.114.011759_TableS3.pdf]

Table S3 The number of differentially expressed splicing junctions. Here a DE junction must be outside DE genes to disambiguate isoform DE from gene DE. There is one order of magnitude of difference between different strains or time points. We took a decidedly conservative approach. There likely are more differentially expressed splicing junctions than we found.

	**MA15**		**PR8**	
**Strain**	**Day 2**	**Day 4**	**Day 2**	**Day 4**
**129S1**	453	326	219	720
**AJ**	25	237	306	174
**B6**	817	183	42	628
**CAST**	855	581	192	783
**NOD**	206	282	521	438
**NZO**	612	184	101	268
**PWK**	692	976	64	235
**WSB**	964	276	75	822
