# Supplementary material for: Genomic Profiling of Collaborative Cross Founder Mice Infected with Respiratory Viruses Reveals Novel Transcripts and Infection-Related Strain-Specific Gene and Isoform Expression
Source: G3 (Bethesda). 2014 Jun 5;4(8):1429–44. doi: 10.1534/g3.114.011759 (PMC4132174; doi:10.1534/g3.114.011759)
Supplement: Supporting Information [file supp_g3.114.011759_TableS4.pdf]

Table S4 The replicate size for each combination of mouse strain, virus, and days-post-infection. Note that mocks are pooled for all DE analysis.

Strain	Virus	Day	Sample size
**129S1**	MA15	2	3
**129S1**	MA15	4	3
**129S1**	MOCK	2	2
**129S1**	MOCK	4	2
**129S1**	PR8	2	3
**129S1**	PR8	4	3
**AJ**	MA15	2	3
**AJ**	MA15	4	2
**AJ**	MOCK	2	2
**AJ**	MOCK	4	2
**AJ**	PR8	2	3
**AJ**	PR8	4	3
**B6**	MA15	2	3
**B6**	MA15	4	3
**B6**	MOCK	2	2
**B6**	MOCK	4	2
**B6**	PR8	2	2
**B6**	PR8	4	3
**CAST**	MA15	2	3
**CAST**	MA15	4	2
**CAST**	MOCK	2	2
**CAST**	MOCK	4	2
**CAST**	PR8	2	3
**CAST**	PR8	4	2
**NOD**	MA15	2	3
**NOD**	MA15	4	3
**NOD**	MOCK	2	2
**NOD**	MOCK	4	2
**NOD**	PR8	2	3
**NOD**	PR8	4	3
**NZO**	MA15	2	2
**NZO**	MA15	4	2
**NZO**	MOCK	2	2
**NZO**	MOCK	4	2
**NZO**	PR8	2	2
**NZO**	PR8	4	2
**PWK**	MA15	2	3
**PWK**	MA15	4	3
**PWK**	MOCK	2	2

**PWK**	MOCK	4	2
**PWK**	PR8	2	2
**PWK**	PR8	4	3
**WSB**	MA15	2	3
**WSB**	MA15	4	3
**WSB**	MOCK	2	2
**WSB**	MOCK	4	2
**WSB**	PR8	2	3
**WSB**	PR8	4	3
