# Supplementary material for: Genomic Profiling of Collaborative Cross Founder Mice Infected with Respiratory Viruses Reveals Novel Transcripts and Infection-Related Strain-Specific Gene and Isoform Expression
Source: G3 (Bethesda). 2014 Jun 5;4(8):1429–44. doi: 10.1534/g3.114.011759 (PMC4132174; doi:10.1534/g3.114.011759)
Supplement: Supporting Information [file supp_g3.114.011759_TableS5.pdf]

Table S5 Viral read counts in the mock-infected founders. Each strain's total comes from four mice. The counts are low, several orders of magnitude lower than infected samples. The fact we still get non-zero reads may be an artifact of mapping and select strains may have partial sequence similarity to viruses.

Strain	MA15	PR8
**129S1**	5084	11394
**AJ**	9549	2659
**B6**	521	1656
**CAST**	10490	3060
**NOD**	608	857
**NZO**	629	636
**PWK**	957	634
**WSB**	144	7491
